# The Efficacy and Safety of Icotinib in Patients with Advanced Non-Small Cell Lung Cancer Previously Treated with Chemotherapy: A Single-Arm, Multi-Center, Prospective Study

**DOI:** 10.1371/journal.pone.0142500

**Published:** 2015-11-24

**Authors:** Xingsheng Hu, Li Zhang, Yuankai Shi, Caicun Zhou, Xiaoqing Liu, Dong Wang, Yong Song, Qiang Li, Jifeng Feng, Shukui Qin, Nong Xv, Jianying Zhou, Li Zhang, Chunhong Hu, Shucai Zhang, Rongcheng Luo, Jie Wang, Fenlai Tan, Yinxiang Wang, Lieming Ding, Yan Sun

**Affiliations:** 1 Department of Medical Oncology, Cancer Institute and Hospital, Chinese Academy of Medical Sciences and Peking Union Medical College, Beijing Key Laboratory of Clinical Study on Anticancer Molecular Targeted Drugs, Beijing, China; 2 Sun Yat-Sen University Cancer Center, Guangzhou, China; 3 Shanghai Pulmonary Hospital, Tongji University, Shanghai, China; 4 Department of Pulmonary Oncology, 307 Hospital of the Academy of Military Medical Sciences, Cancer Center, Beijing, China; 5 Third Affiliated Hospital, The Third Military Medical University of People‘s Liberation Army, Chongqing, China; 6 Nanjing Military General Hospital, Nanjing, China; 7 Changhai Hospital, The Second Military Medical University, Shanghai, China; 8 Jiangsu Province Cancer Hospital, Nanjing, China; 9 Nanjing Bayi Hospital of People’s Liberation Army, Nanjing, China; 10 The First Affiliated Hospital of College of Medicine, Zhejiang University, Hangzhou, China; 11 Peking Union Medical College Hospital, Beijing, China; 12 The Second Xiangya Hospital, Changsha, China; 13 Beijing Chest Hospital, Capital Medical University, Beijing Tuberculosis and Thoracic Tumor Research Institute, Beijing, China; 14 Nanfang Hospital, Southern Medical University, Guangzhou, China; 15 Beijing Cancer Hospital, Beijing, China; 16 Betta Pharmaceuticals Co, Ltd, Hangzhou, China; Catalan Institute of Oncology, SPAIN

## Abstract

**Background:**

Icotinib is a small molecule targeting epidermal growth factor receptor tyrosine kinase, which shows non-inferior efficacy and better safety comparing to gefitinib in previous phase III trial. The present study was designed to further evaluate the efficacy and safety of icotinib in patients with advanced non-small-cell lung cancer (NSCLC) previously treated with platinum-based chemotherapy.

**Methods:**

Patients with NSCLC progressing after one or two lines of chemotherapy were enrolled to receive oral icotinib (125mg tablet, three times per day). The primary endpoint was progression-free survival. The secondary endpoints included overall survival, objective response rate, time to progression, quality of life and safety.

**Results:**

From March 16, 2010 to October 9, 2011, 128 patients from 15 centers nationwide were enrolled, in which 124 patients were available for efficacy evaluation and 127 patients were evaluable for safety. The median progression-free survival and time to progression were 5.0 months (95%CI 2.9–6.6 m) and 5.4 months (95%CI 3.1–7.9 m), respectively. The objective response rate and disease control rate were 25.8% and 67.7% respectively. Median overall survival exceeded 17.6 months (95%CI 14.2 m-NA) according to censored data. Further follow-up of overall survival is ongoing. The most frequent treatment-related adverse events were rash (26%, 33/127), diarrhea (12.6%, 16/127) and elevation of transaminase (15.7%, 20/127).

**Conclusions:**

In general, this study showed similar efficacy and numerically better safety when compared with that in ICOGEN trial, further confirming the efficacy and safety of icotinib in treating patients with advanced NSCLC previously treated with chemotherapy.

**Trial Registration:**

ClinicalTrials.gov NCT02486354

## Introduction

Recent analysis of cancer incidence and mortality demonstrated that lung cancer is the leading cause of the cancer-related death both worldwide and nationwide in China, which highlights the urgent need for more effective therapeutic interventions.[1, 2] First-line platinum-based doublet chemotherapy regimens have achieved 1-year survival rates of approximately 34%. The targeted agents, such as gefitinib, erlotinib, and afatinib, have further improved outcome for patients with a driver mutation in first-line setting with a median PFS around 9.5 to 13.1 months. [3–6] Second-line therapy for advanced NSCLC included single docetaxel, pemetrexed, erlotinib, gefitinib with a tumor response of 6.3–9.1%, and a median PFS of 2.2–6.3 months in unselected population. [7–9]

Icotinib (Betta Pharmaceuticals Co., Ltd, Hangzhou, China) is a potent, orally active, highly selective EGFR-TKI. [10] A favorable safety profile was noted in its phase I and II trials, with rash and diarrhea as the most common adverse events. [11, 12] Based on the phase III trial, icotinib was approved by the China Food and Drug Administration (CFDA) as second- or third-line treatment for advanced NSCLC. [13] ICOGEN is a randomized, double-blind, head-to-head, phase III trial designed to determine whether icotinib is non-inferior to gefitinib in patients with locally advanced or metastatic NSCLC after failure of at least one platinum-based chemotherapy regimen. In this study, icotinib was non-inferior to gefitinib in terms of progression-free survival (HR 0.84, 95% CI 0.67–1.05; median progression-free survival 4.6 [3.5–6.3] months vs. 3.4 [2.3–3.8] months; p = 0.13). [13] The most common adverse events were rash (icotinib vs. gefitinib: 40.5% vs. 49.2%) and diarrhea (21.5% vs. 29.1%). Icotinib was associated with lower rates of drug-related adverse events (60.5% [121/200] vs. 70.4% [140/199]; p = 0.05), especially drug-related diarrhea (18.5% [37/200] vs. 27.6% [55/199]; p = 0.03). [13] A retrospective phase IV study further assessed the efficacy and safety of icotinib, and tumor response was observed across a broad range of patient subtypes with favorable safety profile. [14]

To further explore the role of icotinib as second-line treatment, here we present a single-arm, prospective study to evaluate the efficacy and safety of icotinib in patients with advanced NSCLC previously treated with platinum-based chemotherapy.

## Methods

### Study design and patients

This study was a single-arm, multi-center, prospective trial, aiming to evaluate the efficacy and safety of icotinib in patients with locally advanced or metastatic NSCLC after failure of at least one platinum-based chemotherapy regimen.

Eligible subjects are histologically or cytologically confirmed locally advanced/metastatic (stage IIIB/stage IV, using the American Joint Committee on Cancer [AJCC] 6th edition of tumor-node-metastasis [TNM] staging system) NSCLC patients progressed after at least one platinum-based chemotherapy regimen at entry. Other eligibility criteria included: age 18 to 75 years; Eastern Cooperative Oncology Group (ECOG) performance status (PS) 0–2; at least one measurable lesion by Response Evaluation Criteria in Solid Tumors version 1.0; and adequate hematologic and biochemical values. Patients with symptomatic brain metastases; malignant tumor within the previous five years; severe infection; congestive heart failure; previous treatment with drugs targeting EGFR; and a history of interstitial lung disease were excluded from this study.

The study protocol was approved by independent ethics committees at 15 participating centers in China, the full names of all the ethics committee/institutional review board(s) that approved our study are listed in S1 Appendix. All subjects provided written informed consent prior to participating in the study. The study was undertaken in full accordance with the guidelines for Harmonization/Good Clinical Practice, the Declaration of Helsinki, and other bioethical principles. This study was retrospectively registered as NCT02486354.

### Procedures

Patients were enrolled by physician referral and administered with oral icotinib (tablet form, 125 mg) three times daily by investigators at the 15 participating centers within two days after enrollment until disease progression or unacceptable toxicity. Treatment interruption (no more than 14 days) was recommended when grade 3 or higher adverse events occurred, and dose reduction of icotinib was not permitted. If the adverse events did not resolve after a 14-day interruption, patients withdrew and were excluded from the final data analysis.

The primary endpoint was progression-free survival (PFS) in the full analysis set. Secondary endpoints included overall survival (OS), time-to-progression (TTP), overall response rates (ORR), disease control rate (DCR), toxicity, safety and quality of life (QoL).

PFS was defined as the duration between the date of entering the study and the date of disease progression or death. TTP was defined as the duration between the date of study entry and the date of disease progression. OS was defined as the time between the date of study entry and the date of death from any cause. The efficacy was evaluated by investigators. Tumor response assessment was conducted using RECIST version 1.0 at the 4 week after the first medication, and then at a 6-week intervals. We assessed QoL using the 4th edition of the Functional Assessment of Cancer Therapy-Lung (FACT-L) questionnaires and Lung Cancer Symptoms Subscale (LCS). [15] Toxicities were monitored and graded according to the National Cancer Institute Common Toxicity Criteria for Adverse Events, version 3.0 (CTCAE v3.0). [16]

### Statistical analysis

Since icotinib is a completely new drug, and only 200 patients treated with icotinib were enrolled in the registered phase 3 ICOGEN study, more data were needed for IND application. Therefore, after completing the registered ICOGEN study, China food and drug administration dictated an additional single-arm study of the study drug involving at least 100 subjects in same population to further explore the efficacy and safety of icotinib. This is how the sample size was determined in this study.

Descriptive statistical analysis was performed in this study using SAS 9.1.3. The mean, standard deviation, median, minimum and maximum were used to describe the measurement data. Frequency and percentage were used to describe the categorical data. The ORR was defined as the sum of CR and PR, and DCR was defined as the sum of CR, PR and stable disease (SD). The Kaplan-Meier curve was performed to estimate the median and the 95% CIs for PFS and OS and draw survival curves. The LCS Scale total score and the FACT-L scores were analyzed by covariance analysis. Adverse events (AEs) and drug-related AEs were coded and graded according to CTCAE v3.0. The descriptive summary of the index of laboratory examination mainly aimed at the abnormal data.

Efficacy analysis was performed in the full analysis set (FAS), which included all randomized subjects who have completed at least 1-cycle (28 days as 1 cycle) treatment of the study drug, and safety and toxicity were analyzed in the safety set (SS), including all randomized subjects who have used at least 1 dose of the study drug.

## Results

**Fig 1:**
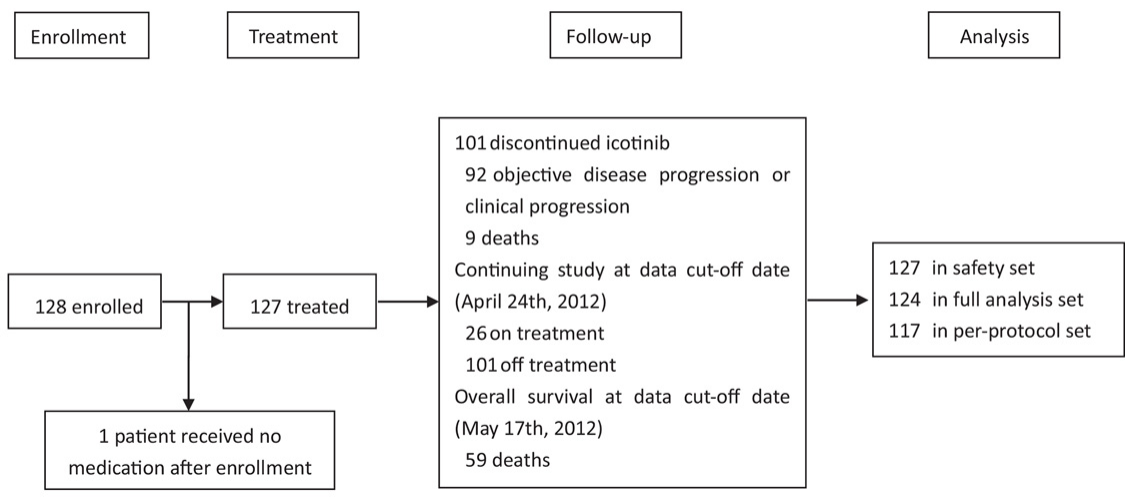
Study profile.

Between March 16, 2010 and October 9, 2011, a total of 128 patients were enrolled in the study (Fig 1), in which 127 were treated and evaluable for safety analysis (1 case was excluded from the safety set [SS] due to no medication after enrollment), and 124 subjects were included in the full analysis set (FAS, 4 subjects were excluded from the FAS due to the informed consent form withdrawal or protocol violation). A total of 117 patients were included in per protocol set (PPS), 11 patients did not complete the study (Table A in S1 Appendix).

### Baseline characteristics

The baseline characteristics are summarized in Table 1. The median age was 57 years (range: 30–73 years), the females and males accounted for 52.4% (n = 65) and 47.6% (n = 59) respectively. The majority of patients had stage IV disease (89.5%, n = 111), and 74.2% (n = 92) had adenocarcinoma. Four patients excluded from the FAS due to consent withdrawal or protocol violation, the baseline characteristics were listed in Table B in S1 Appendix.

**Table 1:** Baseline characteristics (the full analysis set).

Characteristics		Icotinib (n = 124)
Median age (range, years)		57 (30–73)
Sex	Male	65 (52.4%)
	Female	59 (47.6%)
Smoking status	Smokers	51 (41.1%)
	Non-smokers	73 (58.9%)
ECOG PS^a^	0–1	118 (95.9%)
	2	5 (4.1%)
Tumor histology	Squamous-cell carcinoma	25 (20.3%)
	Adenocarcinoma	91 (74.0%)
	Adenosquamous carcinoma	2 (1.6%)
	Other	5 (4.1%)
Disease stage	IIIB	13 (10.5%)
	IV	111 (89.5%)

Data are number (%) of subjects or median (range) of age.

^a^ ECOG = Eastern Cooperative Oncology Group.

### Efficacy

**Fig 2:**
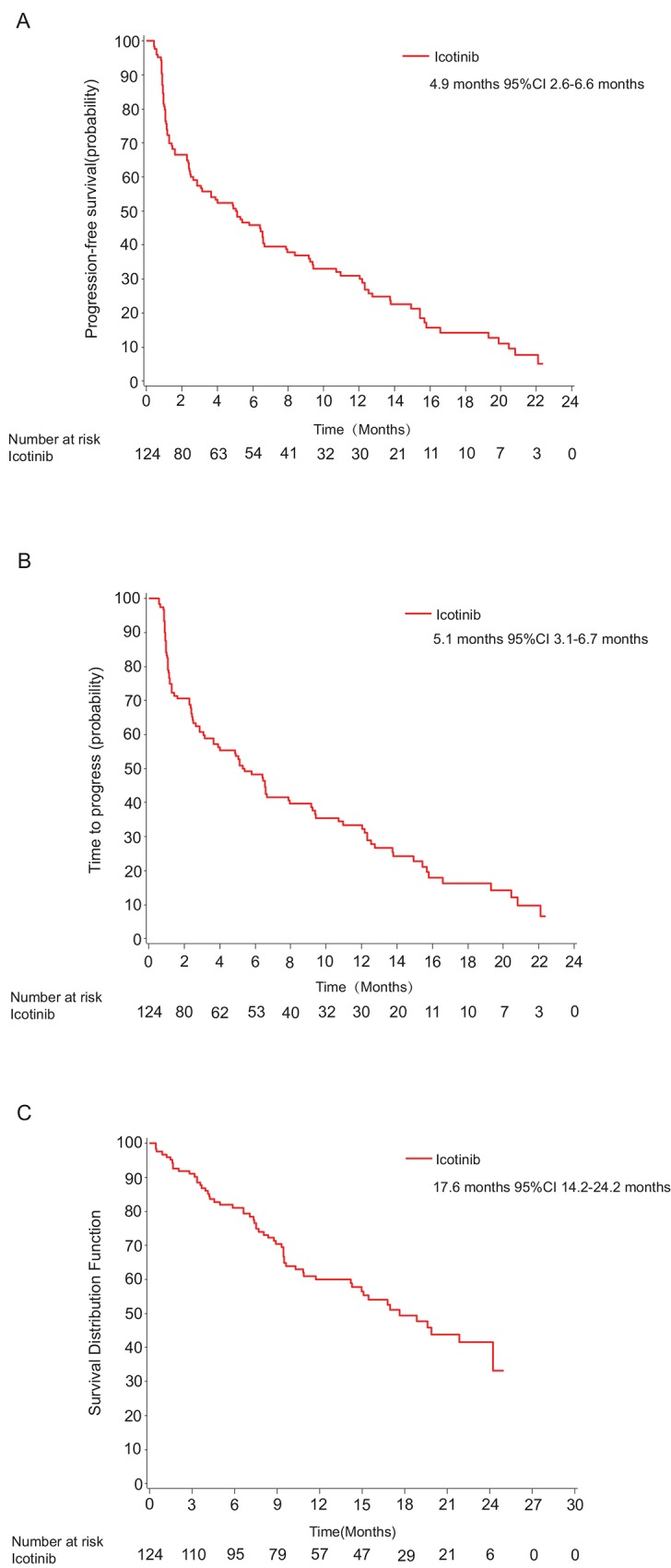
Kaplan-Meier analysis of primary and secondary endpoints in full-analysis set (FAS). Kaplan-Meier curves for progression-free survival (A), time to progression (B), and overall survival (C).

At the cut-off (April 24, 2012), a total of 124 subjects entered the FAS, and 101 (81%) had primary endpoint events (92 disease progression and 9 death). The median PFS and TTP were 5.0 months (95%CI 2.9–6.6 m) and 5.4 months (95%CI 3.1–7.9 m), respectively (Fig 2, Table 2). With respect to other efficacy endpoints, the ORR was 25.8% (32/124), including 32 PRs, and the DCR was 67.7% (84/124) in the FAS population (Table 3). The per-protocol analysis for PFS, TTP, ORR and DCR were listed in Table C in S1 Appendix, and S1 File.

**Table 2:** Tumor response (the full analysis set).

	N (%)
CR	-
PR	32 (25.8%)
SD	52 (41.9%)
PD	33 (26.6%)
Death	5 (4.0%)
Other	2 (1.6%)
ORR	32 (25.8%)
DCR	74 (67.7%)
PFS (month)	5.0 (95%CI 2.9–6.6)
TTP (month)	5.4 (95%CI 3.1–7.9)
OS (month)	NA (95%CI 14.2-NA)

^a^ Other included patients who was not evaluable or tolerable.

**Table 3:** Progression-free survival by subgroup (FAS).

		N	PFS (Median, month)	95% CI (month)	P value
Gender	Male	59	2.4	1.2–3.6	0.0003
	Female	65	9.4	5.8–12.5	
ECOG PS	0–1	119	5.1	2.9–6.7	0.1178
	2	5	3.9	0.8–8.4	
Smoking status	Smokers	73	2.3	1.2–3.6	0.0003
	Non-smokers	51	7.9	5.3–12.3	
Disease stage	IIIB	13	5.1	2.4-NA	0.1420
	IV	111	5.0	2.6–6.6	
Tumor histology	Adenocarcinoma	92	7.9	4.9–11.0	0.0005
	Non-adenocarcinoma	32	1.5	1.1–2.9	
Best response	CR+PR	32	15.4	12.3–19.3	0.0002
	SD	52	5.8	4.0–9.2	

At the OS cut-off (May 17, 2012), 59 cases reached the OS endpoint in FAS with an estimated median OS exceeding 17.6 months (95%CI 14.2–NA) according to censored data. Further follow-up of OS is ongoing.

Subgroup analysis demonstrated that females, adenocarcinoma, non-smokers, and patients with CR/PR had significantly better PFS than males, non-adenocarcinoma, smokers and patients with SD. However, there was no statistically significant difference between different ECOG score and diseases stage (Table 3). The OS analysis also showed similar results.

Multivariate analysis was also performed to detect potential predictors by Cox model. As showed in Table 4, adenocarcinoma, stage IIIb, and non-smokers were the significant variable after a stepwise selection in the model for progression-free survival.

**Table 4:** Summary of multivariate analysis for progression-free survival.

Predictor variables for progression-free survival	Hazard ratio (95% CI)	P value
Men vs women^a^	1.63 [0.89, 2.96]	0.1114
Non-adenocarcinoma vs Adenocarcinoma^b^	2.28[1.38, 3.76]	0.0013
ECOG 0–1 vs 2^a^	0.87 [0.35, 2.19]	0.7670
Stage IV vs IIIb^b^	2.38[1.12, 5.05]	0.0235
Smoker vs non-smoker^b^	1.84[1.21, 2.81]	0.0046

^a^ Results from the full model.

^b^ Results from the model after stepwise selection.

### Safety

The overall adverse events incidence was 70.2% (89/124), with rash (26.6%, 33/124), diarrhea (12.1%, 15/124), and elevation of transaminase (14.5%, 18/124) as the most frequent treatment-related AEs (>10%). Other AEs and drug-related AEs were listed in Table 5. Most AEs were mild (grade I/II) and manageable, only one grade 3 elevation of transaminase and one fatal ILD was seen in the present study.

**Table 5:** Adverse events and drug-related adverse events^a^.

	Adverse events (N = 124)	Drug-related adverse events (N = 124)
Rash	33 (26.6%)	33 (26.6%)
Transaminase elevation	20 (16.1%)	18 (14.5%)
Pain	18 (14.5%)	—
Diarrhea	16 (12.9%)	15 (12.1%)
Hypo-albuminemia	7 (5.6%)	—
Fever	7 (5.6%)	—
Total	87 (70.2%)	61 (49.2%)

^a^ Table 5 listed all adverse events and drug-related adverse events with incidence over 5%. Data are number (%) of subjects, unless otherwise stated. Some patients reported have more than 1 AEs. All adverse events were assessed according to the National Cancer Institute Common Terminology Criteria for Adverse Events (CTCAE, version 3.0).

Four patients were excluded from the FAS due to consent form withdrawal or protocol violation, in which 3 patients suffered at least 1 AE. One had possibly treatment-related leucopenia and neutropenia, one had treatment-unrelated urinary tract infection, and one had treatment-unrelated hypohemoglobinemia, and erythrocytopenia. All these AEs were mild to moderate.

We recorded serious adverse events (SAEs) in 14 patients (11.3%), including 4 deaths and 3 hospitalizations due to disease progression, 4 lung infections, 1 oral ulcer with fungal infection, 1 sudden death, and 1 death related to interstitial lung disease. Most of them were considered as not treatment-related by investigator, the death related to interstitial lung disease was the only possibly treatment-related serious adverse event. No treatment-related discontinuation was recorded.

## Discussion

This phase 3, prospective, single-arm study demonstrated that icotinib provided favorable clinical outcome benefits in pretreated NSCLC patients with a median PFS of 5.0 months, which was consistent with that in the confirmatory phase 3 ICOGEN study (median PFS 4.6 months).[13] In addition, a good safety profile was also observed in this study.

Several studies evaluated the role of icotinib in pretreated NSCLC patients. The phase 3 ICOGEN study compared icotinib with gefitinib in 400 NSCLC patients who had progressed with at least 1 chemotherapy regimen. [13] In this study, icotinib displayed non-inferior efficacy to gefitinib, with the median PFS for icotinib and gefitinib as 4.6 months and 3.4 months, respectively, without statistical significance. [13] A phase 4 study further confirmed these outcomes in a large and heterogenerous patients population. [14] A total of 6,087 patients with advanced NSCLC were registered in this study, of which 5,549 were evaluable for safety and tumor response analysis. The ORR for second- and multiple lines were 30.3% and 30.4% respectively. [14]

The current study was an expansion of the ICOGEN study utilizing same criteria. Similar baseline characteristics were observed between the two studies in icotinib-treated population except that the current study recruited more patients with ECOG PS 0–1 (95.9% vs 86.9%, p = 0.0078, Table D in S1 Appendix), which might contribute to the numerically longer PFS. Results of our efficacy analysis showed that the PFS and OS were 5.0 months and 17.6 months, respectively. The findings were favorably comparable with those in previous studies. Other efficacy endpoints, including TTP (5.1 months), ORR (25.8%) and DCR (67.7%), were also consistent with the results from the ICOGEN study (TTP: 5.2 month; ORR: 27.6%; DCR: 75.4%) [13].

Current therapy for second-line and multiple-line setting included single docetaxel, pemetrexed, erlotinib and gefitinib. Chemotherapies provided an ORR of 6.3–7.6% and a PFS around 2.0–2.7 months.[8,9] EGFR TKIs, such as gefitinib and erlotinib, merited similar outcome benefits to chemotherapy with a PFS of 2.2 months in overall population, and a PFS around 3.0–3.4 months in Asian population.[7–9] The phase 4 Tarceva Lung Cancer Survival Treatment (TRUST) study recruited a total of 6,665 patients worldwide, with 1,242 patients recruited within the East/Southeast (E/SE) Asian region.[17,18] The median PFS for the E/SE Asian population was 5.78 months, compared with 2.92 months for the non-E/SE Asian population and 3.25 months for the overall global population. As a subgroup of Asian patients, 519 Chinese patients were enrolled. Of these patients, one case had complete response, 127 cases had partial response, 263 cases had stable disease, and 88 cases had progressive disease, resulting in a 26.7% ORR and a 75.3% DCR. The median TTP was 6.44 months, and median OS was 15.37 months. [17]

Asian origin, sex, smoking status, and histologic findings help to identify patients who have a higher likelihood of positive EGFR mutation. [5] In this study, females, those with adenocarcinoma, never smokers had significantly better PFS than the males, those with non-adenocarcinoma, and current smokers. In addition, results from the multivariate analysis suggested an association between PFS and adenocarcinoma, stage IIIb disease, and non-smokers.

As suggested by the results of the current study, there was no unexpected safety finding relevant to icotinib. Most drug-related AEs were generally mild to moderate with only 1 reported case of grade 3 transaminase elevation and 1 ILD. Both frequency and severity of drug-related AEs were consistent with the previous work studying icotinib. [11–14]

Interstitial lung disease (ILD) is considered as a major safety concern for EGFR-TKIs. The incidence of ILD among gefitinib-treated patients was 0.44–2.0%, and the mortality rate of ILD was reported to be 0.12–0.5%.[19–21] ILD was also found in patients receiving erlotinib, with the incidence of 0.1–4.8% and the mortality of 0.8–2.4%.[22–26] Several factors were reported to contribute to the development of ILD, including older age, poorer performance status, smoking status, shorter duration since diagnosis of NSCLC, reduced normal lung, preexisting ILD, and concurrent cardiac disease.[19] For icotinib, one ILD case was reported in a phase I study,[11] and no ILDs was reported with icotinib or gefitinib in the ICOGEN study.[13] In the IV phase study, three ILD-like events were observed, and all of these cases were in elderly patients with stage IV adenocarcinoma who had received at least one platinum-based chemotherapy.[14] In the present study, one ILD case was reported who had received two platinum-based chemotherapy regimens. Investigators considered this ILD as icotinib-related by CT scan and clinical syndrome.

In summary, this trial further confirmed efficacy and safety of icotinib in treating patients with advanced NSCLC previously treated with chemotherapy. Icotinib is a potential option for pretreated NSCLC population with favorable antitumor activity and safety profile.

## Supporting Information

S1 TREND Checklist Trend Checklist. (PDF)

S1 Appendix Text. The list of all the ethics committee/institutional review board(s) that approved the present study. Table A. Number of patients included in each analysis set. Table B. Baseline characteristics of the 4 patients excluded from FAS. Table C. Tumor response (the per-protocol set). Table D. Baseline characteristics of the current study and the ICOGEN study.
(DOCX)

S1 File Kaplan-Meier analysis of primary and secondary endpoints in per-protocol set (PPS). Kaplan-Meier curves for progression-free survival (Figure A), time to progression (Figure B), and overall survival (Figure C).
(EPS)

S1 Protocol Trial protocol. (PDF)

## References

[1] Siegel R, Naishadham D, Jemal A. Cancer statistics, 2012. CA Cancer J Clin. 2012 Jan-Feb; 62(1):10–29. 10.3322/caac.20138 22237781

[2] Herbst RS, Heymach JV, Lippman SM. Lung cancer. N Engl J Med. 2008 9 25; 359(13):1367–80. 10.1056/NEJMra0802714 18815398 PMC10662965

[3] Rosell R, Carcereny E, Gervais R, Vergnenegre A, Massuti B, Felip E, et al Erlotinib versus standard chemotherapy as first-line treatment for European patients with advanced EGFR mutation-positive non-small cell lung cancer (EURTAC): a multicentre, open-label, randomised phase 3 trial. Lancet Oncol. 2012 3; 13(3):239–46. 10.1016/S1470-2045(11)70393-X 22285168

[4] Zhou C, Wu YL, Chen G, Feng J, Liu XQ, Wang C, et al Erlotinib versus chemotherapy as first-line treatment for patients with advanced EGFR mutationpositive non-small-cell lung cancer (OPTIMAL, CTONG-0802): a multicentre, open-label, randomised, phase III study. Lancet Oncol. 2011 8; 12(8):735–42. 10.1016/S1470-2045(11)70184-X 21783417

[5] Mok TS, Wu YL, Thongprasert S, Yang CH, Chu DT, Saijo N, et al Gefitinib or carboplatin-paclitaxel in pulmonary adenocarcinoma. N Engl J Med. 2009 9 3; 361(10):947–57. 10.1056/NEJMoa0810699 19692680

[6] Sequist LV, Yang JC, Yamamoto N, O'Byrne K, Hirsh V, Mok T, et al Phase III study of afatinib or cisplatin plus pemetrexed in patients with metastatic lung adenocarcinoma with EGFR mutations. J Clin Oncol. 2013 9 20;31(27):3327–34. 10.1200/JCO.2012.44.2806 23816960

[7] Shepherd FA, Rodrigues Pereira J, Ciuleanu T, Tan EH, Hirsh V, Thongprasert S, et al Erlotinib in previously treated non-small-cell lung cancer. N Engl J Med. 2005 7 14; 353(2):123–32. 16014882 10.1056/NEJMoa050753

[8] Kim ES, Hirsh V, Mok T, Socinski MA, Gervais R, Wu YL, et al. Gefitinib versus docetaxel in previously treated non-small-cell lung cancer (INTEREST): a randomized phase III trial. 2008 Nov 22;372(9652):1809–18. 10.1016/S0140-6736(08)61758-4 19027483

[9] Ciuleanu T, Stelmakh L, Cicenas S, Miliauskas S, Grigorescu AC, Hillenbach C, et al Efficacy and safety of erlotinib versus chemotherapy in second-line treatment of patients with advanced, non-small-cell lung cancer with poor prognosis (TITAN): a randomised multicentre, open-label, phase 3 study. Lancet Oncol. 2012 3;13(3):300–8. 10.1016/S1470-2045(11)70385-0 22277837

[10] Hu S, Xie G, Zhang DX, Davis C, Long W, Hu Y, et al Synthesis and biological evaluation of crown ether fused quinazoline analogues as potent EGFR inhibitors. Bioorg Med Chem Lett. 2012 10; 22(19): 6301–6305. 10.1016/j.bmcl.2012.06.067 22959248

[11] Zhao Q, Shentu J, Xu N, Zhou J, Yang G, Yao Y, et al Phase I study of icotinib hydrochloride (BPI-2009H), an oral EGFR tyrosine kinase inhibitor, in patients with advanced NSCLC and other solid tumors. Lung Cancer. 2011 8; 73(2):195–202. 10.1016/j.lungcan.2010.11.007 21144613

[12] Wang HP, Zhang L, Wang YX, Tan FL, Xia Y, Ren GJ, et al Phase I trial of icotinib, a novel epidermal growth factor receptor tyrosine kinase inhibitor, in Chinese patients with non-small cell lung cancer. Chin Med J (Engl). 2011 7 5;124(13):1933. 22088449

[13] Shi Y, Zhang L, Liu X, Zhou C, Zhang L, Zhang S, et al Icotinib versus gefitinib in previously treated advanced non-small-cell lung cancer (ICOGEN): a randomised, double-blind phase 3 non-inferiority trial. Lancet Oncol. 2013 9;14(10):953–61. 10.1016/S1470-2045(13)70355-3 23948351

[14] Hu X, Han B, Gu A, Zhang Y, Jiao SC, Wang CL, et al A single-arm, multicenter, safety-monitoring, phase IV study of icotinib in treating advanced non-small cell lung cancer (NSCLC). Lung Cancer. 2014 11; 86(2):207–12. 10.1016/j.lungcan.2014.08.014 25261231

[15] Cella DF, Bonomi AE, Lloyd SR, Tulsky DS, Kaplan E, Bonomi P. Reliability and validity of the Functional Assessment of Cancer Therapy-Lung (FACT-L) quality of life instrument. Lung Cancer. 1995 6;12(3):199–220. 7655830 10.1016/0169-5002(95)00450-f

[16] Trotti A, Colevas AD, Setser A, Rusch V, Jaques D, Budach V, et al CTCAE v3.0: development of a comprehensive grading system for the adverse effects of cancer treatment. Semin Radiat Oncol. 2003 7;13(3):176–81. 12903007 10.1016/S1053-4296(03)00031-6

[17] Reck M, van Zandwijk N, Gridelli C, Baliko Z, Rischin D, Allan S, et al Erlotinib in advanced non-small cell lung cancer: efficacy and safety findings of the global phase IV Tarceva Lung Cancer Survival Treatment study. J Thorac Oncol. 2010 10; 5(10):1616–22. 10.1097/JTO.0b013e3181f1c7b0 20736854

[18] Heigener DF, Wu YL, van Zandwijk N, Mali P, Horwood K, Reck M. Second-line erlotinib in patients with advanced non-small-cell lung cancer: subgroup analyses from the TRUST study. Lung Cancer. 2011 11; 74(2):274–9. 10.1016/j.lungcan.2011.02.017 21439671

[19] Tsuboi M, Le Chevalier T. Interstitial lung disease in patients with non-small-cell lung cancer treated with epidermal growth factor receptor inhibitors. Med Oncol. 2006;23(2):161–70. 16720916 10.1385/MO:23:2:161

[20] Cersosimo RJ.Gefitinib: an adverse effects profile. Expert Opin Drug Saf. 2006 5; 5(3):469–79. 16610973 10.1517/14740338.5.3.469

[21] Maemondo M, Minegishi Y, Inoue A, Kobayashi K, Harada M, Okinaga S, et al First-line gefitinib in patients aged 75 or older with advanced non-small cell lung cancer harboring epidermal growth factor receptor mutations: NEJ 003 study. J Thorac Oncol. 2012 9; 7(9):1417–22. 22895139 10.1097/JTO.0b013e318260de8b

[22] Makris D, Scherpereel A, Copin MC, Colin G, Brun L, Lafitte JJ, et al Fatal interstitial lung disease associated with oral erlotinib therapy for lung cancer. BMC Cancer. 2007 8 5;7:150 17683587 10.1186/1471-2407-7-150 PMC1963454

[23] Gemma A. Drug-induced interstitial lung diseases associated with molecular-targeted anticancer agents. J Nippon Med Sch. 2009 2; 76(1):4–8. 19305103 10.1272/jnms.76.4

[24] Cohen MH, Johnson JR, Chen YF, Sridhara R, Pazdur R. FDA drug approval summary: erlotinib (Tarceva) tablets. Oncologist. 2005 Aug;10(7):461–6. 16079312 10.1634/theoncologist.10-7-461

[25] Nguyen KS, Neal JW. First-line treatment of EGFR-mutant non-small-cell lung cancer: the role of erlotinib and other tyrosine kinase inhibitors. Biologics. 2012; 6:337–45. 10.2147/BTT.S26558 23055691 PMC3459550

[26] Yang ZY, Yuan JQ, Di MY, Zheng DY, Chen JZ, Ding H, et al Gemcitabine plus erlotinib for advanced pancreatic cancer: a systematic review with meta-analysis. PLoS One. 2013;8(3):e57528 10.1371/journal.pone.0057528 23472089 PMC3589410

