# Supplementary material for: The Efficacy and Safety of Icotinib in Patients with Advanced Non-Small Cell Lung Cancer Previously Treated with Chemotherapy: A Single-Arm, Multi-Center, Prospective Study
Source: PLoS One. 2015 Nov 24;10(11):e0142500. doi: 10.1371/journal.pone.0142500 (PMC4657981; doi:10.1371/journal.pone.0142500)
Supplement: S1 Appendix — The list of all the ethics committee/institutional review board(s) that approved the present study. Table A. Number of patients included in each analysis set. Table B. Baseline characteristics of the 4 patients excluded from FAS. Table C. Tumor response (the per-protocol set). Table D. Baseline characteristics of the current study and the ICOGEN study. (DOCX) [file pone.0142500.s002.docx]

**Text. The list of all the ethics committee/institutional review board(s) that approved the present study**

1. Ethics Committee of Department of Medical Oncology, Cancer Institute and Hospital, Chinese Academy of Medical Sciences and Peking Union Medical College, Beijing Key Laboratory of Clinical Study on Anticancer Molecular Targeted Drugs

2. Ethics Committee of Sun Yat-Sen University Cancer Center

3. Ethics Committee of Shanghai Pulmonary Hospital, Tongji University

4. Ethics Committee of Department of Pulmonary Oncology, 307 Hospital of the Academy of Military Medical Sciences, Cancer Center

5. Ethics Committee of Third Affiliated Hospital, The Third Military Medical University of People‘s Liberation Army

6. Ethics Committee of Nanjing Military General Hospital

7. Ethics Committee of Changhai Hospital, The Second Military Medical University

8. Ethics Committee of Jiangsu Province Cancer Hospital

9. Ethics Committee of Nanjing Bayi Hospital of People’s Liberation Army

10. Ethics Committee of The First Affiliated Hospital of College of Medicine, Zhejiang University

11. Ethics Committee of Peking Union Medical College Hospital

12. Ethics Committee of The Second Xiangya Hospital

13. Ethics Committee of Beijing Chest Hospital, Capital Medical University, Beijing Tuberculosis and Thoracic Tumor Research Institute

14. Ethics Committee of Nanfang Hospital, Southern Medical University

15. Ethics Committee of Beijing Cancer Hospital

**Table A. Number of patients included in each analysis**

| **Data set** | **Icotinib (n=128) (%)** |  |
| --- | --- | --- |
| **Enrolled** | 128 |  |
| **Safety set** | 127 (99.2%) |  |
| excluded | 1 (0.8%) |  |
| no medication after enrollment | 1 |  |
| **Full analysis set** | 124 (96.9%) |  |
| excluded | 4 (3.1%) |  |
| withdraw the informed consent | 1 |  |
| used the banned drugs before enrollment | 1 |  |
| there is ILD before enrollment | 1 |  |
| no medication after enrollment | 1 |  |
| **Per protocol set** | 117 (91.4%) |  |
| Excluded | 11 (8.6%) |  |
| Informed consent withdrawal  Rejection to continue treatment | 1  3 |  |
| Recieving other treatment | 1 |  |
| Usage of the banned drugs before enrollment | 1 |  |
| ILD history | 1 |  |
| Lost of follow-up | 1 |  |
| No medication after enrollment | 1 |  |
| Poor compliance | 2 |  |

**Table B. Baseline characteristics of the 4 patients excluded from FAS**

| Characteristics | | Icotinib (n=4) |
| --- | --- | --- |
| Median age (range, years) | | 68 (60-69.5) |
| Sex | Male | 3 (75%) |
|  | Female | 1 (25%) |
| Smoking status | Smokers | 1 (25%) |
|  | Non-smokers | 3 (75%) |
| ECOG PS^a^ | 0-1 | 3 (75 %) |
|  | 2 | 1 (25%) |
| Tumor histology | Squamous-cell carcinoma | 1 (25%) |
|  | Adenocarcinoma | 3 (75%) |
| Disease stage | IV | 4 (100%) |
| Number of previous chemotherapy regimen | 1 | 2 (50%) |
|  | 2 or more | 2 (50%) |

^a^ ECOG=Eastern Cooperative Oncology Group

**Table C. Tumor response (the per-protocol set)**

|  | **N (%)** |
| --- | --- |
| CR | - |
| PR | 31 (26.5%) |
| SD | 48 (41.0%) |
| PD | 33 (26.6%) |
| Death | 5 (4.0%) |
| Other | 2 (1.6%) |
| ORR | 31 (26.5%) |
| DCR | 79 (67.5%) |
| PFS (month) | 4.9 (95%CI 2.6-6.6) |
| TTP (month) | 5.1 (95%CI 3.1-6.7) |
| OS (month) | 17.6 (95%CI 14.2 -24.2) |

^a^ Other included patients who was not evaluable or tolerable.

**Table D. Baseline characteristics of the current study and the ICOGEN study**

| Characteristics | | Single-arm study (n=124) | ICOGEN study-icotinib group (n=199) [13] | P value |
| --- | --- | --- | --- | --- |
| Median age (range, years) | | 57 (30-73) | 57 (28-75) | NA |
| Sex | Male | 65 (52.4%) | 117 (58.8%) | 0.2613 |
|  | Female | 59 (47.6%) | 82 (41.2%) |  |
| Smoking status | Smokers | 51 (41.1%) | 98 (49.3%) | 0.1547 |
|  | Non-smokers | 73 (58.9%) | 101 (50.7%) |  |
| ECOG PS | 0-1 | 118 (95.9 %) | 173 (86.9%) | 0.0078 |
|  | 2 | 5 (4.1%) | 26 (13.1%) |  |
| Tumor histology | Squamous-cell carcinoma | 25 (20.3%) | 34 (17.1%) | 0.1381 |
|  | Adenocarcinoma | 91 (74.0%) | 149 (74.9%) |  |
|  | Adenosquamous carcinoma | 2 (1.6%) | 0 (0.0%) |  |
|  | Other | 5 (4.1%) | 16 (8.0%) |  |
| Disease stage | IIIB | 13 (10.5%) | 37 (18.6%) | 0.0501 |
|  | IV | 111 (89.5%) | 162 (81.4%) |  |
