# Supplementary material for: The Efficacy and Safety of Icotinib in Patients with Advanced Non-Small Cell Lung Cancer Previously Treated with Chemotherapy: A Single-Arm, Multi-Center, Prospective Study
Source: PLoS One. 2015 Nov 24;10(11):e0142500. doi: 10.1371/journal.pone.0142500 (PMC4657981; doi:10.1371/journal.pone.0142500)
Supplement: S1 Protocol — (PDF) [file pone.0142500.s004.pdf]

Clinical Study Protocol

A randomized, double-blind, double-dummy, parallel-controlled multicenter phase II/III trial to evaluate the safety and efficacy of Icotinib and Gefitinib in advanced metastatic patients with NSCLC previously treated with chemotherapy

(ICOGEN)

Protocol No.:	BD-IC-III01
Version:	Version 1.2
Date:	July 2, 2009

Sponsor: **Zhejiang Beta Pharma Inc.**

CRO: **Hangzhou Tigermed Consulting Co., Ltd**

Confidentiality Statement

All information contained within the protocol is the property of Zhejiang BetaPharma Co., Ltd, and is therefore only for review by the investigators, co-investigators, ethnics committee and regulatory authorities. Without a prior written permission of Zhejiang BetaPharma Co., Ltd, except during signing an informed consent form with subjects and offering necessary explanations, disclosure of any information to a third party unrelated to the present trial is strictly prohibited.

Sponsor

Company name	Zhejiang Beta Pharma Inc		
Project director	Tan Fen-lai		
Contact address	Building C10-1, BDA International Enterprise Avenue, No. 2 North Jingyuan Street, Beijing Yizhuang Economic Development Zone	Postal code	100176
Telephone	010-67869692-607	Mobile	15801648388
Fax	010-67869291	E-mail	fenlai.tan@betapharma.com.cn

CRO

Company name	Hangzhou Tigermed Consulting Co., Ltd		
Project director	Hu Zhen-yu		
Contact address	Room 813, Huawen Plaza, No. 999 Zhongshan Road West, Shanghai	Postal code	200051
Telephone	86-21-32503704-805	Mobile	13917389217
Fax	86-21-32503707.	E-mail	zhenyu.hu@tigermed.net

Data management

Company name	Hangzhou Tigermed Consulting Co., Ltd		
Project director	Xiao Fang-qiu		
Contact address	Room 813, Huawen Plaza, No. 999Zhongshan Road West, Shanghai	Postal code	200051
Telephone	86-21-32503704-835	Mobile	13916348130
Fax	86-21-32503707	E-mail	fangqiu.xiao@tigermed.net

Statistical analysis unit

Company name	Hangzhou Tigermed Consulting Co., Ltd		
Statistician	Wei Zhao-hui		
Contact address	Room 813, Huawen Plaza, No. 999 Zhongshan Road West, Shanghai	Postal code	200051
Telephone	86-21-32503704-836	Mobile	13661901298
Fax	86-21-32503707	E-mail	zhaohui.wei@tigermed.net

Central Lab: Cancer Institute and Hospital, Chinese Academy of Medical Sciences

<table>
<tr><td>Lab name</td><td colspan="4">Lab of Internal Medicine, Cancer Institute and Hospital, CAMS</td></tr>
<tr><td>Project director</td><td colspan="4">Han Xiao-hong</td></tr>
<tr><td>Address</td><td colspan="2">No. 17 Panjiayuannanli,
Chaoyang District, Beijing</td><td>Postal code</td><td>100021</td></tr>
<tr><td>Telephone</td><td>010-67781331-8448</td><td>Mobile</td><td colspan="2">13810659230</td></tr>
<tr><td>Fax</td><td></td><td>E-mail</td><td colspan="2">xiaohonghan@hotmail.com</td></tr>
</table>

List of Investigators

Principal investigator	Sun Yan, Cancer Institute and Hospital, Chinese Academy of Medical Sciences (CAMS)		
Center No.	01		
Address	No. 17 Panjiayuannanli, Chaoyang District, Beijing		
Postal code	100021	Telephone	010-87788495

Principal co-investigator	Zhang Li, Sun Yat-sen University Cancer Center		
Center No.	02		
Address	No. 651 East Dongfeng Road, Guangzhou		
Postal code	510060	Telephone	13902282893

Co-investigator	Shi Yuankai, Cancer Institute and Hospital, Chinese Academy of Medical Sciences (CAMS)		
Center No.	01		
Address	No. 17 Panjiayuannanli, Chaoyang District, Beijing		
Postal code	100021	Telephone	13701251865

Co-investigator	Zhang Li, Peking Union Medical College Hospital		
Center No.	03		
Address	No. 41 Damucang Hutong, Xicheng District, Beijing,<br>Peking Union Medical College Hospital		
Postal code	100032	Telephone	13121432126

Co-investigator	Wang Jie, Beijing Cancer Hospital		
Center No.	04		
Address	No. Fucheng Road, Haidian District, Beijing		
Postal code	100036	Telephone	010-88196660

Co-investigator	Zhang Shu-cai, Beijing Hospital for Chest Tumors & Tuberculosis Diseases		
Center No.	05		
Address	No. 97 Machang, Tongzhou District, Beijing		
Postal code	101149	Telephone	13901297065

Co-investigator	Liu Xiao-qing, No. 307 Hospital of Chinese PLA		
Center No.	06		
Address	No. 6 East Avenue, Fengtai District, Beijing		
Postal code	100071	Telephone	13681187500

Co-investigator	Jiao Shun-chang, General Hospital of Chinese PLA		
Center No.	07		
Address	No. 28 Fuxing Road, Beijing		
Postal code	100853	Telephone	13801380677

Co-investigator	Liang Li, Peking University Third Hospital		
Center No.	08		
Address	No. 49 North Garden Road, Beijing		
Postal code	100091	Telephone	13241870816

Co-investigator	Dai Hong, Beijing Chaoyang Hospital		
Center No.	09		
Address	No. 8 South Gongti Road, Chaoyang District, Beijing,<br>Beijing Chaoyang Hospital		
Postal code	100020	Telephone	13621205353

Co-investigator	Zhou Cai-cun, Shanghai Municipal Lung Hospital		
Center No.	10		
Address	No. 507 Zhengmin Road, Yangpu District, Shanghai,<br>Shanghai Municipal Lung Hospital		
Postal code	200433	Telephone	13301825532

Co-investigator	Jin Ye-ning, Affiliated Ruijin Hospital, Shanghai Jiao Tong University		
Center No.	11		
Address	No. 573 Xujiahui Road, Shanghai Ruijin Hospital, Shanghai		
Postal code	200023	Telephone	13501634131

Co-investigator	Bai Chun-xue, Affiliated Zhongshan Hospital, Fudan University		
Center No.	12		
Address	Floor 5, Inpatient Building No. 8, No. 180 Fenglin Road, Shanghai		
Postal code	200032	Telephone	13681971807

Co-investigator	Li Qiang, Affiliated Changhai Hospital, Second Miilitary Medical University of Chinese PLA		
Center No.	13		
Address	Building No. 11 (Medical Inpatient), No. 174 Changhai Road,<br>Yangpu District, Shanghai		
Postal code	200433	Telephone	13801602220

Co-investigator	Wu Yi-long, Guangdong Provincial People’s Hospital		
Center No.	14		
Address	Weilun Building, Tumor Center Lung Division,<br>No. 106 Second Zhongshan Road, Guangzhou		
Postal code	510080	Telephone	82837812-21187

Co-investigator	Luo Rong-cheng, Nanfang Hospital of Nanfang Medical University		
Center No.	15		
Address	Floor 6, Building of Internal Medicine,<br>No. 1838 North Guangzhou Avenue, Guangzhou		
Postal code	510515	Telephone	13902251269

Co-investigator	Zhang Wei-min, General Hospital of Guangzhou Military Command of PLA		
Center No.	16		
Address	No. 111 Liuhua Road, Yuexiu District, Guangzhou		
Postal code	510000	Telephone	13380052360

Co-investigator	Qin Shu-kui, No. 81 Hospital of Chinese PLA		
Center No.	17		
Address	Zone 9, No. 34 Block 34 Yanggongjing, Nanjing, Jiangsu		
Postal code	210002	Telephone	13905158713

Co-investigator	Feng Ji-feng, Jiangsu Cancer Hospital		
Center No.	18		
Address	No. 42 Baiziting, Nanjing		
Postal code	210009	Telephone	13901581246

Co-investigator	Song Yong, General Hospital of Nanjing Military Command of PLA		
Center No.	19		
Address	Department of Respiratory Medicine, No. 305 East Zhongshan Road, Nanjing, Jiangsu		
Postal code	210002	Telephone	13851761392

Co-investigator	Zhou Jian-ying, and Xu Nong, First Affiliated Hospital of Medical School of Zhejiang University		
Center No.	20		
Address	Department of Respiratory Medicine, Floor 14,<br>No. 79 Qingchun Road, Hangzhou		
Postal code	310003	Telephone	13505719970

Co-investigator	Zhang Yi-ping, Zhejiang Cancer Hospital		
Center No.	21		
Address	No. 38 Guangji Road, Banshanqiao, Hangzhou		
Postal code	310022	Telephone	13750881678

Co-investigator	Hu Chun-hong, Second Xiangya Hospital, South Central University		
Center No.	22		
Address	No. 139 Middle Renmin Road, Changsha, Hunan		
Postal code	410011	Telephone	13508486908

Co-investigator	Chen Jian-hua, Hunan Cancer Hospital		
Center No.	23		
Address	No. 582 Xianjiahu Road, Changsha, Hunan		
Postal code	410013	Telephone	13807486627

Co-investigator	Wang Dong, Third Affiliated Hospital, Third Military Medical University		
Center No.	24		
Address	No. 10 Yangtze River Road, Daping, Yuzhong District, Chongqing		
Postal code	400042	Telephone	13708280988

Co-investigator	Zhang He-long, Tangdu Hospital of Fourth Military Medical University of Chinese PLA		
Center No.	25		
Address	Department of Oncology, No. 1 Xinsi Road, Baqiao District, Xi’an		
Postal code	710000	Telephone	13519128910

Co-investigator	Liu Wen-chao, Xijing Hospital of Fourth Military Medical University of Chinese PLA		
Center No.	26		
Address	No. 15 West Changle Road, Xi’an		
Postal code	710032	Telephone	13991801698

Co-investigator	Chen Ying, Jilin Cancer Hospital		
Center No.	27		
Address	Department of Medcine I, No. 1018 Huguang Road, Changchun, Jilin		
Postal code	130012	Telephone	0431-85871236

Synopsis

**Protocol No.**	BD-IC-III01
**Protocol title**	A randomized, double-blind, double-dummy, parallel-controlled multicenter phase II/III trial to evaluate the safety and efficacy of Icotinib and Gefitinib in advanced metastatic patients with NSCLC previously treated with chemotherapy
**VersionNo./ Date**	Version 1.2/July 2nd, 2009
**Sponsor**	Zhejiang Beta Pharma Inc.
**Clinical trial phase**	Phase III
**Indication**	Advanced non-small cell lung cancer (NSCLC)
**Objectives**	**Primary objective:**<br>To compare the PFS of icotinib versus gefitinib in NSCLC patients<br>**Secondary objective:**<br>To compare the OS, ORR, DCR, TTP, HRQoL, and the safety of icotinib with gefitinib in treatmenting NSCLC patients;<br>**Exploratory objective:** To examine the correlation of EGFR and K-ras gene mutations with efficacy.
**Trial design**	Randomized, double-blind, double-dummy, parallel controlled, multi-center and phase II/III trial
**Number of subjects**	Randomized controlled trial: test group: n=200, control group: n=200;<br>Single-arm trial in test group: n=100.
**Number of study sites**	27
**Study schedule**	The patients may receive treatment with icotinib or gefitinib until progression disease or intolerable toxicity. At a monthly enrollment rate of 2-3 patients per center, enrollment will take around 8-10 months. The entire trial duration will be around 15-18 months. The trial is planed to start in Febuary 2009. The enrollment will end in October-December

	2009, and the entire study will end in August-October 2010.
**Target population**	The target population includes patients aged 18-75 years old with an life expectancy of at least 12 weeks. Subjects are histologically or cytologically confirmed locally advanced (stage IIIB) or metastatic (stage IV) NSCLC patients who must have progressed after at least one platinum-based chemotherapy regimen. Previous one or two chemotherapeutic regimens are also needed, of which at least one must be platinum-based. The previous chemotherapy and the present trial registration must be at least 4 weeks apart. And they must have recovered from any toxicity of a previous chemotherapy. Those who have received only one radio-sensitizing dose of platinum-based regimen are not eligible for enrollment.<br>Inclusion criteria:<br>The subjects must fulfill all the following inclusion criteria to be eligible for participation in the present trial:<br>1. Histologically or cytologically confirmed local advanced or metastatic stage IIIB/IV PD NSCLC not suitable for radical operation or radiotherapy;<br>2. They must have progressed after at least one chemotherapeutic regimen, of which at least one must be platinum-based. The previous chemotherapy and the present trial registration must be at least 4 weeks apart. And they must have recovered from any toxicity of a previous chemotherapy;<br>3. Subjects who have received only one radio-sensitizing dose of platinum will not be eligible for enrollment;<br>4. Aged 18-75 years old;<br>5. Based upon the RECIST criteria 1.0, at least one measurable and non-irradiated lesions is required:<br>● At least one measurable lesion is required. If only one lesion is present, the nature of the neoplasm at the lesion must be confirmed cytologically and/or histologically;<br>● Accurately measurable lesion with single diameter equal to or greater than 20 mm with conventional techniques (chest or abdomen CT or MRI), or equal to or greater than 10 mm with spiral CT.

	6. ECOG performance score (PS): 0-2;<br>7. Life expectancy of at least 12 weeks;<br>8. Adequate organ function meeting the following:<br>● Bone marrow: ANC ≥1.5×10^9/L (1500/mm^3); platelet ≥ 75×10^9/L; hemoglobin ≥9 g/dL;<br>● Liver: Serum bilirubin ≤ 2 ×ULN，AST and ALT ≤ 2.5 ×ULN (if hepatic metastasis exist, permitting AST&ALT≤5 ×ULN);<br>● Kidney: Cr ≤1.5 ×ULN;<br>● No malabsorption or other gastrointestional diseases interfering with drug absorption;<br>9. Within 7 days prior to the start of therapy, women of child-bearing potential must undergo a pregnancy test, which must be negative; men of child-bearing potential: contraceptive measures must be adopted during surgical sterization and within 3 months afterward;<br>10. Subjects who understand and voluntarily signed a written informed consent form.<br>Exclusion criteria:<br>A subject fulfilling any of the following exclusion criteria is not eligible for participation in this trial:<br>1. Usage of Iressa, Tarceva, Erbitux and Herceptin for anti-neolasmic therapy prior to enrollment;<br>2. Concurrent use of phenytoin sodium, carbamazepine, rifampicin, barbital or St. John's Wort;<br>3. Known severe hypersensitivities to icotinib or any one of the following vehicles (tablet core excipients: lactose, microcrystalline cellulose, croscarmellose sodium, polyvinylpyrrolidone K30 and magnesium stearate; coating agent excipients: titanium dioxide, hydroxypropyl methyl cellulose and sedian);<br>4. Use of unapproved drugs or other study drugs within 4 weeks prior to the date of the fisrt trial medication;<br>5. Patient organ system status:<br>● Patients with brain metastasis despite previous diagnosis & therapy, undergoing no operation and/or radiotherapy; unstable

	clinical status requiring symptomatic therapy; no hormone is needed, but spaced from symptomatic therapy less than 4 weeks;<br>● Bone marrow-metastasized patients;<br>● A history of instistial pulmonary disease, drug-induced interstitial disease, radiation pneumonitis requiring hormonal therapy or active ILD with any clinical evidence;<br>● Idiopathic pulmonary fibrosis on baseline CT scan;<br>● Based upon the investigator's judgment, presence of severe or impossible-to-control systemic diseases (e.g. unstable respiratory, cardiac, hepatic or renal disease);<br>● Any unstable systemic disease (including active infection, Grade IV hypertension, unstable angina pectoris, congestive heart failure, hepatic/renal or metabolic disease);<br>● Any other malignant tumor (excluding completely cured cervical in situ carcinoma or basal cell/squamous epithelial cell skin cancer) within the last 5 years;<br>● Previous definite diagnosis of neuropsychiatric disturbances, including epilepsy or dementia;<br>6. Patient organ lesions at functional levels:<br>● Bone marrow: ANC <1.5 $\times 10^9$/L (1500/mm^3), platelet <75 $\times 10^9$ /L or hemoglobin <9 g /dl;<br>● Liver: serum bilirubin > 2 ×ULN2. When there is no hepatic metastasis, ALT/AST > 2.5 ×ULN (hepatic metastasis > 5 ×ULN);<br>● Kidney: Cr > 1.5 ×normal standard value;<br>● Presence of any other important clinical abnormality or lab examination test result rendering a patient not eligible for trial participation;<br>● Based upon Common Terminology Criteria for Adverse Events (CTCAE) version 3.0 (NCI-CTCAE 3.0) > Grade II chronic toxicity (excluding hair loss) ongoing.<br>7. Patients previously registering to accept treatment with the present trial or withdrawing from the present trial are not eligible for re-enrollment;<br>8. Sumultaneously accepting any other anti-neoplastic therapy;

	9. Pregnant or lactating women;<br>10. In the opinion of investigator, a subject is unlikely to complete the present trial or comply with the trial's requirements (due to management or other causes).
**Study drug**	Experimental group: Icotinib 125 mg tablets, po, 1# tid and gefitinib 250 mg dummy tablets, po, 1# qd until PD or an intolerable toxicity.<br>Control group: Gefitinib 250 mg tablets, po, 1# qd and icotinib 125mg dummy tablets, po, 1#, tid until PD or an intolerable toxicity.
**Concomitant medication**	No use of systemic therapy or radiotherapy known to be effective for NSCLC is allowed during the course of therapy. Refer to protocol section 10.2 for detailed specifications on other concomitant medications.
**Assignment**	Once the eligibility of a subject is confirmed, the investigator logs onto the randomization system, enters his or her relevant information and obtains a randomization code by which to dispense the relevant drug. The subjects will be randomized into experimental or control group by stratification factors include smoking status, pathologic types and PS score; randomization adopts the minimization method.<br>A patient must start treatment within 24-48h after randomization.<br>No repeated randomization is adopted for the present trial.
**Trial procedures**	After signing the informed consent form and fulfilling the inclusion/exclusion criteria, the present trial shall proceed with dynamic randomization. The distribution ratio of experimental group and control group is 1:1. A total of 400 subjects will be enrolled (experimental group: n=200, control group: n=200).<br>In the light of the fact that the sample size of experimental group has yet to reach the minimal of 300 cases as recommended by the SFDA guideline for phase III clinical trials, after completing the originally scheduled sample size of 200 pairs, the official response of SFDA dictates a further single-arm study of the study drug involving at least 100 subjects.

	For evaluations, a patient is required to come for visits at Day 1, Day 28 (Week 4) and then at 6-week enternals until PD or intolerable toxicity or other specific situation fulfilling the definition in section 4.3. Patients discontinued from study treatment for any cause other than progression (except for patient withdrawing informed consent) should continue receiving tumor assessments every 6 weeks until PD.<br>After documentation of PD, the investigator should contact the patient, his/her family members or the current attending physician by telephone at least once every 8 weeks to collect the information on survival.
**Efficacy variables**	Primary efficacy variable: PFS.<br>Secondary efficacy variable: Secondary efficacy variables include OS, ORR, DCR, TTP and lung cancer symptoms and health-related quality-of-life (HRQoL).<br>Evaluation parameters of exploratory study:<br>EGFR and K-ras gene mutations.
**Safety variables**	AE, vital signs and laboratory parameters.
**Statistical analysis**	Refer to section. 14 Statistics for details

Contents

LIST OF ABBREVIATIONS OR SPECIAL TERMS.. **1**

1 TRIAL BACKGROUND 4

1.1 OVERVIEW OF LUNG CANCER 4

1.2 DRUG OVERVIEW 5

1.3 STUDY RATIONALE 7

2 OBJECTIVES... 7

2.1 PRIMARY OBJECTIVE 7

2.2 SECONDARY OBJECTIVES 8

2.3 EXPLORATORY OBJECTIVES.. 8

3 EXPECTED TRIAL DURATION, NUMBER OF STUDY SITES AND PLANNED NUMBER OF SUBJECTS 8

4 SELECTION AND WITHDRAWAL OF SUBJECTS.. 8

4.1 INCLUSION CRITERIA... 9

4.2 EXCLUSION CRITERIA 10

4.3 WITHDRAWAL CRITERIA..11

4.3.1 Withdrawal time and methods...11

4.3.2 Withdrawal procedures 11

4.3.3 Replacements of discontinued subjects 12

4.3.4 Follow-up of discontinued subjects...12

5 TRIAL DESIGN 13

5.1 TRIAL TYPE AND DESIGN RATIONALE 13

5.2 TARGET POPULATION: 13

5.3 INTERIM ANALYSIS:...13

5.4 STUDY SCHEDULE: 14

6 RANDOMIZATION METHOD...14

6.1 RANDOMIZATION METHOD:...14

6.2 BLINDING PROCEDURES OF DOUBLE-BLIND TRIAL 14

6.3 UNBLINDING PROCEDURES DURING A DOUBLE-BLIND TRIAL.......................................14

7 STUDY PLAN AND PROCEDURES (REFER TO TABLE 1 FOR FLOWCHART)................15

7.1 SCREENING 15

7.2 EVALUATION DURING TREATMENT...16

7.3 POST-TREATMENT FOLLOW-UPS: 18
7.4 UNSCHEDULED VISITS 19

8 BIOMARKERS FOR EXPLORATORY STUDY AND POPULATION PK STUDY 23

9 STUDY DRUG 23

9.1 NAME, PHYSICOCHEMICAL PROPERTIES, DESCRIPTION, STRENGTH, COMPOSITION, USAGE, PACKAGING AND STORAGE OF STUDY DRUG 23
9.2 NAME, PHYSICOCHEMICAL PROPERTIES, DESCRIPTION,STRENGTH, COMPOSITION, USAGE, PACKAGING AND STORAGE OF CLINICAL CONTROL DRUG 24
9.3 DRUG PACKAGE AND LABELING 26
9.4 DRUG DISPENSING AND INVENTORY 26

10 SUBJECT THERAPY 26

10.1 STUDY DRUG 26
10.2 CONCOMITANT MEDICATION 27
10.2.1 Prohibited medication prior to trial 27
10.2.2 Permitted and prohibited concomitant medications during the study 27
10.3 SUBJECT COMPLIANCE 28

11 EFFICACY EVALUATION 29

11.1 PRIMARY EFFICACY VARIABLE 29
11.2 SECONDARY EFFICACY VARIABLES 30

12 SAFETY ASSESEMENT 30

12.1 SAFETY VARIABLES 30
12.1.1 Vital signs and physical examination 30
12.1.2 Laboratory examinations 30
12.2 ADVERSE EVENTS 31
12.2.1 Definition of AE 31
12.2.3 Unexpected AEs 32
12.2.4 Observation, recording and reporting of AEs 32
12.2.5 Evaluation for AE severity 33
12.2.6 Relationship between AEs and study drug 33
12.2.7 Treatment, follow-up and duration of AEs 34
12.2.8 Common AEs of icotinib and gefitinib 34
12.2.9 Determination and handling of laboratory abnormalities 35
12.3 SAES 35
12.3.1 Defintion of SAE 35

12.3.2 Important AE *36*
12.3.3 Recording and reporting procedures of SAE / pregnancy *36*
12.3.4 Special treatments for SAEs *36*

13 DATA MANAGEMENT **37**

13.1 DATA TRACEABILITY, AND CRF COMPLETION AND TRANSFER 37
13.2 DESIGN AND ESTABLISHMENT OF DATABASE 37
13.3 DATA CODING 37
13.4 DATA ENTRY AND REVISION 37
13.5 DATA VERIFICATION 37
13.6 DATABASE LOCK AND UNBLINDING 38

14 STATISTICS **38**

14.1 ESTIMATION OF SAMPLE SIZE 38
14.2 DEFINITION AND SELECTION OF ANALYSIS SETS 39
14.3 HYPOTHESIS TEST 39
14.4 STATISTICAL METHODS 40
14.4.1 Analytic methods *40*
14.4.2 Interim analysis *41*

15 TRIAL MANAGEMENT **42**

15.1 STATEMENT 42
15.2 ETHNICS CONSIDERATIONS 42
15.3 SOURCE DATA VERIFICATION 42
15.4 QUALITY CONTROL AND ASSURANCE 43
15.5 INFORMED CONSENT FORM/DATA PROTECTION AGREEMENT 43
15.6 REVISION OF CLINICAL PROTOCOL 43
15.7 CRF 43
15.8 TRIAL MONITOR 44
15.9 SUBJECT PRIVACY 44
15.10 PUBLICATIONS 44
15.11 FILING 44

16 REFERENCES **45**

17. PROTOCOL SIGNATURE PAGE **46**

List of abbreviations or special terms

Below is a list of abbreviations or special terms used in the protocol:

**Abbreviation or special term**	**Interpretation**
ADR	Adverse drug reaction
AE	Adverse event
ALP	Alkaline phosphatase
ALT	Alanine transaminase
ANC	Absolute neutrophil count
AST	Aspartate transaminase
AUC	Area under curve
BSA	Body surface area
BSC	Best supportive care
BUN	Blood urea nitrogen
CI	Confidence interval
Cm	Centimeter
CR	Complete response
CRF	Case report form
CT	Computed tomography
CTC	Common Toxicity Criteria
DCR	Disease control rate
EDTA	Ethylenediaminetetraacetic acid
EGFR	Epidermal growth factor receptor
EORTC	European Organization for Research and Treatment of Cancer
EWB	Emotional well-being
FACT-L	Functional Assessment for Cancer Therapy-Lung
FWB	Functional well-being
GCP	Good Clinical Practice
HCG	Human chorionic gonadotropin
HIV	Human immunodeficiency virus
ICH	International Conference on Harmonization of Technical Requirements for Registration of Pharmaceuticals for Human Use
IDMC	Independent data monitoring committee

Abbreviation or special term	Interpretation
IEC	Independent ethics committee
ILD	Interstitial lung disease
INR	International normalized ratio
ITT	Intention-to-treat population
Kg	Kilogram
LD	Longest diameter
LCS	Lung cancer scale
M	Meter
Mg	Milligram
Min	Minute
Ml	Milliliter
Mm	Millimeter
MRI	Magnetic resonance imaging
NCI	National Cancer Institute
NSCLC	Non-small cell lung cancer
ND/NA	Non-detected or not applicable
OAE	Other significant adverse event
OS	Overall survival
ORR	Overall response rate
PFS	Progression-free survival
PD	Progressive disease
PK	Pharmacokinetics
PP	Per-protocol population
PR	Partial response
PRO	Patient reported outcomes
PS	Performance status
QoL	Quality of life
RECIST	Response Evaluation Criteria in Solid Tumors
SAE	Severe adverse event
SD	Stable disease

Abbreviation or special term	Interpretation
SWB	Social/family well-being
TOI	Trial outcome index
TTP	Time to progression
ULN	Upper limit of normal range
WHO	World Health Organization

1 Trial background

1.1 Overview of lung cancer

Lung cancer is the most common death-causing malignancy worldwide. Each year the global number of new cases is estimated at over 1.4 million, and the count of death surpasses 1.2 million. In Asia-Pacific region, the annual number of new cases approaches 500,000 and that of death cases is over 400,000. According to the WHO estimation, the annual number of newly discovered lung cancer patients is over 400,000 in China. And around 317,000 (80%) are non-small-cell lung cancer (NSCLC). The number of death cases stands at around 272,000.

Surgery may provide the greatest probability of cure for the patients with lung cancer, including NSCLC. However, over 50% of the patients have local advanced cancer or distant metastasis, which are not suitable for surgical resection. Platinum-based regimen is currently the most widely used first-line chemotherapy in the world, which could boost the 1-year survival from 25% to 35% and increase the median survival by 1.5 months comparing with best supportive care.

After the administration of first-line chemotherapy, the recurrent or progressive patients require second-line therapy. Although the optimal protocol has remained elusive, the globally recommended second-line chemotherapeutic agents include Taxotere (docetaxol), Alimta (pemetrexed) or epidermal growth factor receptor-tyrosine kinase inhibitors (EGFR-TKIs), e.g. gefitinib and erlotinib. Erlotinib and gefitinib belong to the targeted anti-neoplasmic drugs and their adverse responses are obviously lower than those of docetaxol and pemetrexed, which fall into the category of cytotoxicity with marked adverse events. As demonstrated in several trials, the above second-line therapies could extend the median survival by 1-1.5 months in comparisons with best supporting care. EGFR TKIs as second-line therapy have comparable efficacy, but better safety profiles and offer more marked symptomatic improvement compared with chemotherapy. According to the results of a global clinical trial (INTEREST) (including China), the median OS and PFS for gefitinib and Taxotere were close to each other without statistical significance, while the profiles of AE and quality of life for the former were obviously better than those for the latter.

1.2 Drug overview

Icotinib hydrochloride (tradename: Conmana^{TM}) is an oral edidermal growth factor tyrosine kinase inhibitors (EGFR-TKIs). Belonging to the category of National Class 1.1 New Drug, its chemical structure is similar to gefitinib (Iressa, AstraZencea Inc.) and erlotinib (Tarceva, Roche Pharmaceutical). The international and domestic autonomous intellectual property rights, including US patent, belong to Zhejiang Beta Pharma Inc. EGFR TKIs can suppress the growth of tumor cells, therefore dramatically boost a patient's survival quality without bone marrow and hemopoietic suppressions associated with traditional chemotherapy.

Currently, erlotinib and gefitinib are approved for second or third-line therapies of patients with advanced NSCLC in China. In pre-clinical studies, icotinib hydrochloride displayed potent anti-tumor activity in vitro and in vivo. Moreover, icotinib showed high specificity and selectivity to its target EGFR in a preclinical kinase profiling study, which demonstrated that EGFR and its mutants were solely inhibited among 88 kinases profiled. At the molecular level, the IC50 value of EGFR inhibition for icotinib hydrochloride was 4.5 nM, compared with with 2.5 nM for erlotinib and 27-33 nM for gefitinib. In acute toxicological animal tests, no marked toxicity was observed even at a dose of 8000 mg/Kg BW. And in long-term toxicological experiments in animals, the most common adverse reactions included hair loss and diarrhea. Few animals experienced transient impairments of hepatic functions which disappeared without any intervention.

The phase I clinical trial approval document (2006L01414) was awarded in June 2006. After the start of phase I clinical trial in October 2006, the following trials have been completed:

1. Phase I, single-center, randomized, double-blind and placebo-controlled trial for evaluating the safety and tolerance of single-dose oral icotinib hydrochloride tablets in Chinese healthy male volunteers;

2. Phase I, single-center, open-label, triple-cross trial for evaluating the PK profiles of icotinib hydrochloride tablets in Chinese healthy male volunteers;

3. Phase I, single-center, open-label trial for evaluating the effects of food intake upon the PK profiles of icotinib hydrochloride tablets in Chinese healthy male volunteers;

4.Phase I, single-center and open-label trial for evaluating the profiles of safety, tolerance

and PK of t oral icotinib hydrochloride tablets wice daily for 28 consecutive days in Chinese patients with NSCLC;

5. Phase I, open-label, single-center trial for evaluating the profiles of safety, tolerance and PK of oral icotinib hydrochloride tablets thrice daily for 28 consecutive days in patients with various solid tumor;

6. Phase IIa trial for evaluating the efficacy and safety of continuous dosing in patients with NSCLC.

The results of phase I trials showed icotinib had an excellent safety profile. In healthy subjects, no dose limited toxicity (DLT) was observed even at a single maximal dose of 1025 mg. High-caloric food could enhance the absorption of icotinib hydrochloride. At a daily dose of 100-350 mg, all PK parameters demonstrated a linear relationship. With a half-life of around 6h, it was suitable for an oral administration before or after meal twice or thrice daily.

In advanced NSCLC patients with refractory disease after at least one platinum-based regimen, phase I trials showed that the most common AE was skin rash after multiple dosing for 28 consecutive days, and diarrhea and nausea came next. A great majority of these AEs were mild (Grade I/II). No drug withdrawal or other clinical interventions were needed. Few patients might have an onset of elevated transaminases and disappeared by itselves without discontinuing medication.

According to phase I dose-escalating study, the safety and preliminary efficacy for the patients with NSCLC, colon cancer and breast cancer, were confirmed. The phase IIa study showed that the ORR surpassed 30% in the effective dose group of patients with NSCLC (ITT population). One CR case was found at Peking Union Medical College Hospital and 2 CR cases at First Affiliated Hsopital of Zhejiang University. With respect to safety, the incidence of skin rash and diarrhea was < 50% and <20% respectively, most of which were mild.

The above trial results were submitted to the SFDA-CDE (State Food and Drug Administration, Center for Drug Evaluation, China) in January and September, 2008. In December 2008, a phase II/III clinical trial approval document (2008L11932) was issued by SFDA.

1.3 Study rationale

The non-inferiority trial design is adopted to compare the efficacy and safety of icotinib versus gefitinib in treating patients with advanced NSCLC. The basic design rationale are based upon the results of the above phase I & IIa clinical trial indicating that icotinib hydrochloride may have comparable efficacy and safety with gefitinib.

Belonging to the family of EGFR-targeting tyrosine kinase inhibitors (TKIs), gefitinib is currently in wide applications as second or third-line therapy for patients with NSCLC in China. With definite efficacy, its safety profiles are superior to the standard second-line therapeutic agents, such as Taxotere (docetaxol) and Alimta (pemetrexed). However, as a patented drug imported from foreign countries, gefitinib is rather expensive. And icotinib hydrochloride has been a domestically self-developed product. So if its non-inferiority to gefitinib in terms of efficacy and safety is confirmed, icotinib hydrochloride has potential values of clinical application in China.

The administration of icotinib is also based upon the above trials. The results showed that 300 mg/day (150 mg bid), 300 mg/day (100 mg tid) and 375 mg/day (125 mg tid) are all safe and effective. However, in comparison with 300 mg/day (150 mg bid or 100 mg tid), 375 mg/day (125 mg tid) had similar profiles of adverse reactions but a better efficacy. Furthermore, the corresponding pharmacokinetic studies also showed that all PK parameters (homeostatic blood drug concentration and exposure value) of 375 mg/day (125 mg tid) dose level were markedly higher than those of 300 mg/day (150 mg bid) and 300 mg/day (100 mg tid) dose levels. Therefore, 375 mg/day (125 mg tid) is recommended in this study.

Based upon the above considerations, gefitinib is selected as a positive control for the present trial. According to the non-inferiority trial design, the trial is conducted to evaluate the efficacy and safety of oral icotinib (tablet, 125mg tid) in treating local advanced or mestastasized NSCLC patients who progressed after at least one chemotherapy regimen.

2 Objectives

2.1 Primary objective

To compare the PFS of icotinib with gefitinib in the treatment of patients with NSCLC.

2.2 Secondary objectives

To compare the OS of icotinib with gefitinib in the treatment of patients with NSCLC;

To compare the ORR of icotinib with gefitinib in the treatment of patients with NSCLC;

To compare the DCR of icotinib with gefitinib in the treatment of patients with NSCLC;

To compare the TTP of icotinib with gefitinib in the treatment of patients with NSCLC;

To compare the symptoms improvement and HRQoL of icotinib with gefitinib in the treatment of patients with NSCLC;

To compare the safety of icotinib with gefitinib in the treatment of patients with NSCLC.

2.3 Exploratory objectives

To explore the relations between EGFR, K-ras gene mutations with efficacy.

3 Expected trial duration, number of study sites and planned number of subjects

The patients will receive icotinib or gefitinib until PD or intolerable toxicity. At a monthly enrollment rate of 2-3 patients at each center, the enrollment period is around 8-10months. The observation period is around 6-8 months for the study treatment with icotinib or gefitinib. Therefore the total trial period is around 15-18 months. The present trial will start in Febuary 2009 and enrollment will end during in October-December 2009. The entire trial will end in August-October, 2010.

It is planned that the present trial will recruit 400+100 eligible subjects. A total of 27 centers will participate in the trial nationwide. In stage I, a total of 400 cases will be enrolled (experimental group, n=200; control group, n=200). To fulfill the inquiry comment of SFDA, the experimental group will include another 100 cases in the stage II as single-arm study.

4 Selection and withdrawal of subjects

The study population will be those aged 18-75 years, with life expectancy at least 12 weeks, histologically or cytologically confirmed local advanced or metastatic phase IIIB/IV NSCLC who are not suitable for radical surgery or radiotherapy. They must progress after at least one chemotherapy regimens, of which at least one must be platinum-based. The previous chemotherapy and the present trial registration must be at least 4 weeks apart. And patients must have recovered from any toxicity of a previous

chemotherapy. Those who accepting only one radio-sensitizing dose of platinum-based drug are not eligible for enrollment.

4.1 Inclusion criteria

The subjects must fulfill all the following inclusion criteria to be eligible for participation in the present trial:

1. Histologically or cytologically confirmed local advanced or metastatic stage IIIB/IV PD NSCLC, not suitable for radical operation or radiotherapy;
2. They must progress after at least one chemotherapy regimens, of which at least one must be platinum-based; the previous chemotherapy and the present trial registration must be at least 4 weeks apart. And they must have recovered from any toxicity of a previous chemotherapy;
3. Subjects who have received only one radio-sensitizing dose of platinum will not be eligible for enrollment;
4. Aged 18-75 years old;
5. Based upon the RECIST criteria, there is at least one measurable and non-irradiated lesions:

- There is at least one measurable lesion. If only one lesion is present, the nature of the neoplasm at the lesion must be confirmed cytologically and/or histologically;
- Accurately measurable lesion with single diameter equal to or greater than 20 mm with conventional techniques (chest or abdomen CT or MRI), or equal to or greater than 10 mm with spiral CT.

6. ECOG performance score (PS): 0-2;
7. Life expectancy at least 12 weeks;
8. Adequate organ function meeting the following:

- Bone marrow: ANC $\geq 1.5\times10^9$/L (1500/mm^3), platelet $\geq 75\times10^9$/L and hemoglobin ≥9 g/dL;
- Liver: Serum bilirubin ≤2 ×ULN，AST and ALT ≤2.5 ×ULN (if hepatic metastasis, permitting AST&ALT≤5 ×ULN);
- Kidney: Cr ≤1.5 ×ULN;
- No malabsorption or other gastrointestional diseases interfering with drug absorption;

9. Within 7 days prior to the start of therapy, women of child-bearing potential must undergo a pregnancy test, which must be negative; men of child-bearing potential: contraceptive measures must be adopted during surgical sterization and within 3 months afterward;

10. Subjects who understand and voluntarily signed a written informed consent form.

4.2 Exclusion criteria

A subject fulfilling any of the following exclusion criteria is not eligible for trial participation:

1. Prior to enrollment, use of Iressa, Tarceva, Erbitux and Herceptin for anti-neolasmic therapy;

2. Concurrent use of phenytoin sodium, carbamazepine, rifampicin, barbital or St. John's Wort;

3. Known severe hypersensitivities to icotinib or any one of the following vehicles (tablet core excipients: lactose, microcrystalline cellulose, croscarmellose sodium, polyvinylpyrrolidone K30 and magnesium stearate; coating agent excipients: titanium dioxide, hydroxypropyl methyl cellulose and sedian);

4. Use of unapproved drugs or other study drugs within 4 weeks prior to the fisrt study drug medication;

5. Patient organ system status:

- Patients with brain metastasis despite previous diagnosis & therapy, undergoing no specific operation and/or radiotherapy; symptomatic disease requiring hormonal therapy; no hormone is needed, but spaced from previous hormonal therapy less than 4 weeks;
- Patients with bone marrow metastasis;

6. A history of instistial pulmonary disease, drug-induced interstitial disease, radiation pneumonitis requiring hormonal therapy or active ILD with any clinical evidence; Idiopathic pulmonary fibrosis on baseline CT scan;

7. Based upon the investigator's judgment, presence of severe systemic diseases (e.g. unstable or non-compensated respiratory, cardiac, hepatic or renal disease);

8. Any unstable systemic disease (including active infection, Grade IV hypertension, unstable angina pectoris, congestive heart failure, hepatic/renal or metabolic disease);

9. Within the last 5 years, suffering any other malignant tumor (excluding completely cured cervical in situ carcinoma or basal cell/squamous epithelial cell skin cancer);

10. Previous definite diagnosis of neuropsychiatric disturbances, including epilepsy or dementia.

11. Patient organ lesions at functional levels:

- Bone marrow: ANC <1.5×10^9 /L (1500/mm^3), platelet <75×10^9 /L or hemoglobin <9 g /dl;
- Liver: serum bilirubin > 2 ×ULN2. When there is no hepatic metastasis, ALT/AST > 2.5 ×ULN (hepatic metastasis > 5 ×ULN);
- Kidney: Cr > 1.5 ×normal standard value;
- Presence of any other important clinical abnormality or lab examination test result rendering a patient not eligible for trial participation;
- Based upon Common Terminology Criteria for Adverse Events (CTCAE) version 3.0 (NCI-CTCAE 3.0) > Grade II chronic toxicity (excluding hair loss) ongoing.

12. Patients previously registering to accept treatment with the present trial or withdrawing from the present trial are not eligible for re-enrollment;

13. Sumultaneously accepting any other anti-neoplastic therapy;

14. Pregnant or lactating women;

15. In the opinion of investigator, the subject is unlikely to complete the present trial or comply with the trial's requirements (due to management or other reasons).

4.3 Withdrawal criteria

4.3.1 Withdrawal time and methods

A patient may withdraw from the study treatment and evaluation at any stage. The reason for withdrawals may be as follows:

1. Voluntary withdrawal: A patient may withdraw from the trial at any time without compromising further treatment;
2. In the opinion of the investigator, Beta Pharma and/or Tigermed, there is any safety issue (AE);
3. In the opinions of investigator and/or BetaPharma and/or Tigermed, the patient has a rather poor compliance to study protocol;
4. Death;
5. Patient lost to follow-up;
6. In the opinion of other investigators, the situation justifies study withdrawal.

4.3.2 Withdrawal procedures

For any patient who has withdrawn from the study, the investigator must inquire about

the reason for withdrawal and whether or not there is an occurrence of any AE. If possible, the investigator should visit and assess the discontinued patient. Reason for withdrawal and the last dosing date must be recorded on the CRF. The patient should return the unused study drug.

At the time of study discontinuation, the patient should be further examined if there is an onset of new or worsening abnormal laboratory resultss of CTC grade 3/4. The results should be recorded into the CRF until the results recover to CTC grade 1/2, except where improvement of the measurement is not possible due to the disease itself. For these cases, the investigator should enter his or her comments on the CRF and medical record.

All study-related toxicities and SAEs ongoing at the time of study interruption must be followed up until relieved, unless in the opinion of the investigator, the improvement of this condition is impossible due to the patient's existing condition.

After discontinuation of study treatment, the investigator must track all existing or new AEs occurring within 30 days from the date that the last dose of study drug was given. All new AEs and SAEs occurring during this period should be reported, and SAEs must be reported within 24h to both Tigermed and BetaPharma and tracked similarly until relieved. When a patient is discontinued from the study, the investigator should immediately inform Tigermed and BetaPharma. For any SAE, Tigermed and BetaPharma should be contacted on time following the SAE reporting procedure.

4.3.3 Replacements of discontinued subjects

Patients who are prematurely discontinued from the study will not be replaced.

4.3.4 Follow-up of discontinued subjects

A patient who has discontinued study treatment for any cause other than progression (except for withdrawing informed consent) should continue to receive tumor assessments once every 6 weeks until PD.

After documentation of PD, the investigator should make telephone contacts with the patient, patient's family members or the current attending physician at least once every 8 weeks to collect the information for survival.

5 Trial design

5.1 Trial type and design rationale

This is a randomized, double-blind, double-dummy, parallel-controlled, phase II/III and multi-center trial. The total number of enrolled patients will be around 500 cases. At stage I, 400 cases will be enrolled (experimental group, n=200; control group, n=200). In the regulatory registration process of State Food and Drug Administration (SFDA), based upon the consulting comments of Department of Drug Registration and Center for Drug Evaluation (CDE) of SFDA, it may be necessary to plan a stage II single-arm trial recruiting at least 100 subjects for the test group to fulfill the guideline recommendation of at least 300 cases for the experimental group.

Experimental group:

Icotinib 125 mg tablets, po, #1, tid; gefitinib 250 mg placebo, po, #1, qd until PD or an intolerable toxicity.

Control group:

Icotinib 125 mg placebo, po, #1, tid; gefitinib 250 mg tablets, po, #1, qd until PD or an intolerable toxicity.

Method of administration:

Both gefitinib and dummy tablets are taken before breakfast. And icotinib and dummy tablets are taken #1 q8h.

5.2 Target population:

The patients who have progressed disease or relapsed despite previously received platinum-based chemotherapy regimens. Dynamic randomization is adopted for the present study. Subjects will be randomized into experimental group or control group by stratification factors include smoking status, pathological type and PS score; minimization method of randomization will be adopted.

The patients shall receive icotinib or gefitinib treatment until PD or intolerable toxicity or the occurrence of other specific situation defined under section 4.3.1.

5.3 Interim analysis:

There is no interim analysis.

5.4 Study schedule:

Table 1 presents the number and schedule of planned visits. All investigators should adhere to the trial schedule and procedures and follow the protocol specifications to conduct examinations or evaluations. After screening, each assessment should be conducted within a window of 7 days before or after the scheduled visit date.

6 Randomization method

6.1 Randomization method:

Dynamic randomization will be employed for the present trial. Tigermed Biostatistics will provide a randomization table to generate 400 randomization numbers through a computer program of SAS9.13. The ratio between test group and control group is 1:1 as randomly assigned. These random numbers are reproducible. The block length and seed are recorded into blind code. After sealing, the files are maintained at major study sites and sponsor site. Also they are provided to the central unit of dynamic randomization.

Another cooperating unit of the present trial, Department of Medical Statistics of Fourth Military Medical University, will provide the Interactive Web Response System (IWRS) for this trial. Once the eligibility of a subject is confirmed, the investigators may log in the IWRS randomization system and enter the relevant information, then get the drug number of this subject. Then the corresponding drug may be administered according to the drug number. Based upon the stratification factors, such as smoking status (yes/no), pathologic type (adenocarcinoma/non-adenocarcinoma) and PS score (0-1/2), the subjects are randomized into experimental group or control group. The method of randomization is the Pocock & Simon’s minimal randomization.

The subjects must begin the study treatment within 24-48 hours post-randomization.

Randomization will not be repeated for this trial.

6.2 Blinding procedures of double-blind trial

Based upon the randomization table (blind code), a statistician and staffs unrelated to the study will be in charge of drug packaging and coding. Emergency envelopes are produced simultaneously with the generation of blind code and stored at each center to be used in case of emergent unblinding.

6.3 Unblinding procedures during a double-blind trial

Two unblindings will be performed for the present trial.

First unblinding: After data locking, the staff of blind code maintaining unit, sponsor and statistical staff will cooperate to determine whether the dosing code of a subject is group A or group B for the purpose of statistical analysis.

Second unblinding: statistical unit submits a Statistical Analysis Report. Upon its completion, the drug corresponding to group A/B will be known.

Emergency unblinding: In the events of emergency or SAE, if the investigator deems unblinding necessary, he/she may open and review the subject's corresponding emergency envelope to determine the specific dosing status. While unblinding, the investigator should provide a written explanation of causes and notify the sponsor and monitor.

7 Study plan and procedures (Refer to Table 1 for flowchart)

7.1 Screening

Visit 1(Day –21 to Day 0)

1. Signing written informed consent;
2. Demographic data;
3. Medical history (concurrent non-tumor disease & its concurrent therapy, smoking history, and surgery history) ;
4. Tumor assessment: cranial CT/MRI，bone scan/XR, gadolinium-enchanced spinal MRI, chest and upper abdominal (including bilateral adrenal glands) , enhanced CT-scan, chest XR)
5. Recording AEs and concomitant medications (started immediately after obtaining informed consent);
6. Biomarker sampling: 15 ml whole blood for assay of EGFR/K-ras gene mutation;

Note: Cranial, bone marrow and spinal images are acquired only for the patients with symptoms. If bone scan reveals a new abnormal location, a XR is needed for confirmation (the same evaluation rationale will be followed during the course of therapy).

Day -7 to Day 0

1. Inquiring and recording lung cancer history (major diagnosis, previous anti-tumor therapies or drugs, and metastatic site at baseline);
2. Physical examinations: HR, BP, RR, temperature, height, BW, neurological examinations and ECOG performance status score;
3. Laboratory examinations: hematology: hemoglobin, WBC count, ANC and platelet count;
4. Blood biochemical examinations: TB, AST/ALT, albumin, TP, ALP, BUN, serum Cr and electrolytes (sodium, potassium & calcium)
5. Urine analysis: pH value, urinary glucose, urinary protein, ketone body, occult blood, urobilirubin, erythrocyte and WBC;
6. Only for randomized patients: Tumor tissues samples (paraffin blocks or slides, at least 10 slides for 5 μm thickness tissues, or at least 5 slides for 10 μm thickness tissues)

7.2 Evaluation during treatment

Visit 2 (Week 0/Day 1)

1. Confirming whether or not a subject fulfills the inclusion/exclusion criteria in the study protocol;
2. Conducting physical examinations (HR, BP, RR, temperature, BW, neurological examinations and ECOG performance status score) ;
3. Laboratory examinations (hematology/blood biochemistry/urinary analysis) ;
4. Randomization and dispensation of study drugs;
5. Recording AEs and concomitant medications since the last visit;
6. PRO-Fact L/PRO-LCS questionnaire ;
7. Appointing the next visit;

Visit 3 (Week 4 ±7 days)

1. Conducting physical examinations (HR, BP, RR, temperature, BW, neurological examinations and ECOG performance status score);
2. Laboratory examinations (hematology/blood biochemistry/urinary analysis); ECG/pregnancy trial (as clinically indicated);
3. 2-ml whole blood samples for population PK study;

4. Tumor assessment: cranial CT or MRI, bone scan/XR, gadolinium-enchanced spinal MRI，chest and upper abdomin (including bilateral adrenal glands) enhanced CT-scan & chest XR;
5. Collecting the unused study drug and package, and distributing study drug for next course of treatment;
6. Recording AEs and concomitant medications since the last visit;
7. PRO-FactL/PRO-LCS questionnaire
8. Appointing the next visit;

Visit 4 (Week 10 ±7 days)

1. Conducting physical examinations (HR, BP, RR, temperature, BW, neurological examinations and ECOG performance status score);
2. Laboratory examinations (hematology/blood biochemistry/urinary analysis); ECG/pregnancy trial (as clinically indicated);
3. 2-ml whole blood samples for population PK study;
4. Tumor assessment: cranial CT or MRI, bone scan/XR, gadolinium-enchanced spinal MRI，chest and upper abdomin (including bilateral adrenal glands) enhanced CT-scan & chest XR;
5. Collecting the unused study drug and package, and distributing study drug for next course of treatment;
6. Recording AEs and concomitant medications since the last visit;
7. PRO-FactL/PRO-LCS questionnaire;
8. Appointing the next visit;

Visit 5 (Week 16 ±7 days) and others (visit once every 6 weeks)

1. Conducting physical examinations (HR, BP, RR, temperature, BW, neurological examinations and ECOG performance status score);
2. Laboratory examinations (hematology/blood biochemistry/urinary analysis); ECG/pregnancy trial (as clinically indicated);
3. Tumor assessment: cranial CT or MRI, bone scan/XR, gadolinium-enchanced spinal MRI，chest and upper abdomin (including bilateral adrenal glands) enhanced CT-scan & chest XR;

4. Collecting the unused study drug and package, and distributing study drug for next course of treatment;
5. Recording AEs and concomitant medications since the last visit;
6. PRO-FactL/PRO-LCS questionnaire;
7. Appointing the next visit;

End-of-study/Discontinuation visit

1. Conducting physical examinations (HR, BP, RR, temperature, BW and neurological examinations and ECOG performance status score);
2. Laboratory examinations (hematology/blood biochemistry/urinary analysis); ECG and Pregnancy test;
3. Tumor assessment: cranial CT or MRI, bone scan/XR, gadolinium-enchanced spinal MRI, chest and upper abdomin (including bilateral adrenal glands) enhanced CT-scan & chest XR;
4. Collecting the unused study drug and package from last time;
5. Recording AEs and concomitant medications since the last visit;
6. PRO-FactL/PRO-LCS questionnaire.

7.3 Post-treatment follow-ups:

For patients discontinued study treatment for any cause other than progression (except for patients withdrawing informed consent):

1. Tumor assessment: cranial CT/MRI, bone scan/XR, gadolinium-enchanced spinal MRI, chest and upper abdomin (including bilateral adrenal glands);
2. Recording antitumor therapies or other concomitant medications since the last visit.

Note: If the patient has already received other antitumor therapy, then the patient will no longer receive tumor assessment. However survival data will be extended until death.

For other participating patients:

1. Recording antitumor therapies or other concomitant medications since the last visit;

2. Survival assessment will be extended until death.

7.4 Unscheduled visits

Unscheduled visits may be conducted as clinically indicated. Laboratory abnormalities and AEs with clinical significance should be recorded on the CRF and source documents. If multiple laboratory examinations are conducted within the same day, it is sufficient to record only one set of examination values on the CRF. However all abnormalities of repeated laboratory examinations should be recorded on the CRF.

Table 1. Study schedule

	Screening		Treatment period					End of treatment m	Post-treatment follow-up k
Week (window of ±7 days)			Week 0	Week 4	Week 10	Week 16	Week 22 & others	N/A	N/A
Day	Day -21 to Day 0	Day -7 to Day 0	1	Day 28±7Day	Day 70±7	Day 112±7	Day 154±7 & others		
Visit	1		2	3	4	5	6 & others		7＋
Informed consent	×								
Demographics	×								
Medical & surgical history/smoking history a	×								
Previous radiotherapy & chemotherapy a		×							
Biomarker specimen b	×b								
Population PK s				×	×				
Pregnancy test c		×	As clinically indicated						
Inclusion/exclusion criteria		×							
Physical examinations		×d	×d	×e	×e	×e	×e	×e	
ECOG score		×	× d	×	×	×	×	×	
Concomitant medication f		×	×	×	×	×	×	×	×f
ECG		×	As clinically indicated						
Hematology/blood biochemistry/urine analysis g		×d	×d	×	×	×	×	×	
Tumor assessment h	×			×	×	×	×	×h	×h
PRO -Fact L/ PRO-LCS questionnaire i			×	×	×	×	×	×	×i
Toxicity evaluation/AE j	×	×	×	×	×	×	×	×	
Survival & anti-neoplastic therapy k									×k
Study drug dispensing/use/collection l			×	×	×	×	×	×	

Footnotes

a. A complete medical and surgical history should be obtained. Inquiries should focus on the history of previous and current pulmonary diseases and/or lung-involving systemic disease (e.g. connective tissue diseases). Only during the screening period is the patient smoking status inquired.

b. Blood samples (15ml whole blood) are collected for the assay of EGFR/K-ras gene mutation. In addition, tumor tissue paraffin blocks (tumor tissue must take up at least 1/3 of whole paraffin block) or tumor lesion tissue paraffin slides (not less than 10 slides for 5 μm thickness tissue, or not less than 5 slides for 10 μm thickness tissue) are collected for the randomized subjects. 2ml blood samples for population PK study will be collected during visits 3 and 4.

c. Within 7 days prior to the first dose, women of child-bearing potential must be negative for a urine or serum pregnancy test. If there is a positive result, they are not eligible for trial participation. If pregnancy is suspected during the course of trial, the test should be repeated.

d. If the listed inclusion/exclusion criteria (if applicable) are assessed and fulfilled within 7 days prior to randomization, it is not necessary to repeat them on the first day of dosing unless the investigator thinks that marked changes have occurred.

e. Physical examinations include HR, BP, RR, temperature, height, BW and neurological examinations. If measured during screening, BW may not be repeated. During screening and when clinically indicated, BW will be measured.

f. The concomitant medication data, including drug dose, route of administration, dosing schedule, start date, indication, end date and reason for termination, will be collected from the ongoing of randomized treatment with study drug to 1 month after discontinuation. By learning the patient’s routine prescription information, the attending physician will follow the changes of concomitant medication within the last 6 weeks to evaluate whether or not the improvements of lung symptoms are clinically significant.

g. Within 7 days before randomization, hematology (HB, PLT, WBC, ANC) /blood biochemistry (ALT, AST, ALP, TB, TP, ALB, CR, BUN, K, Na, Ca)/urinalysis will be performed. The subsequent hematology, blood biochemistry and urinalysis should be completed at each visit, 5 days before and after study discontinuation, and when clinically indicated.

h. It is at the discretion of each investigator to determine the imaging method to be adopted. During screening and evaluation, however, various aspects of the disease must be fully defined and the measurable lesions be documented in accordance with the RECIST. Later repeated imaging studies should be conduced once every 6 weeks in accordance with the RECIST guideline, which requires the same evaluation methodology and techniques for every identifiable, and reported lesion during screening and follow-ups. No extra scan is needed to differentiate CR and PR. For a new suspected lesion, proper imaging studies should be conducted. The recommended imaging modalities include enhanced CT scan of chest and upper abdomen (including bilateral adrenal glands), enhanced brain CT/MRI (for symptomatic patients), chest XR, bone scan (if revealing a new abnormal location, disease site should be confirmed by XR) and gadolinium-enchanced spinal MRI (for those with suspected spinal compression). All patients discontinued for causes other than PD should continue objective tumor assessments once every 8 weeks to collect the PD information, unless the patient withdraws informed consent.

i. Patient self-rated questionnaires of FACT-L and LCS version 4 should be completed independently by the patient at the beginning of each visit. The surveys are conducted prior to the patient-physician contacts to ensure that any patient-physician communication will not affect the answering of questionnaire questions. Baseline questionnaire survey should be conducted before the first dose. And subsequent questionnaires will be completed at each visit and at the end of study treatment.

j. In the events of any self-reported ocular symptoms, new or worsening of respiratory symptoms (e.g. cough and wheezing), the patient should seek appropriate medical assistance immediately. Any symptom will be handled according to the clinical routines. If conforming to the definition, it should be reported as AE or SAE. AE collection will start since the patient signs informed consent form. At the end of the study, all ongoing AEs or SAEs should be tracked until resolution unless the investigator thinks that the inherent disease of a patient is unlikely to be relieved. All new AEs and SAEs occurring within 30 days of the last trial dose should be reported. SAEs should be reported within 24h to Tigermed. And tracking is conducted likewise until resolution. During each treatment course, the most severe grade of toxicity is recorded on the CRF.

k. Any patients discontinued from study treatment for any cause other than progression (except for patients withdrawing informed consent) should continue tumor assessments once every 6 weeks to collect PD information. After documentation of PD, the investigator should make telephone contacts with the patient, patient’s family members, or attending physician at least once every 6 weeks to obtain the relevant information on patient OS and post-trial therapy until patient death. During the course of each visit, disease status should be obtained, and all anti-tumor therapies and death dates will be recorded (if applicable).

l. A 4-week supply of study drugs will be dispensed at Visit 2. During subsequent visits, it will be dispensed once every 6 weeks. The unused study drugs and empty packages should be returned. The use status of study drug should be recorded on the CRF, including cause of dose reduction (if applicable).

m. The end-of-treatment visit should be complete within 7 +/-5 days of study discontinuation due to causes other than death or loss to follow-up.

8 Biomarkers for exploratory study and population PK study

As demonstrated by many studies, the efficacy of TKIs was closely correlated with the EGFR and K-ras mutations in the NSCLC patients. The efficacy of the subjects with EFGR mutations of exons 18-21 in ATP-binding functional domain was markedly superior to those of the wild-type subjects. And K-ras gene mutation resulted in the drug resistance to TKIs. Through the genotypic detections of EGFR and K-ras in patient tumor cells, the present trial is conduced to further analyze the EGFR and K-ras mutations and decipher their correlations with the efficacy of icotinib.

For eligible patients screened and randomized, tumor lesion tissue paraffin blocks or slides, if available, will be collected for biomarker analysis. In light of the fact that tumor blocks or slides may not be available from some patients, 15ml blood samples will be drawn from all subjects (including those from whom blocks or slides are collected) for the genotypic detections of EGFR and K-ras during the screening period.

9 Study drug

9.1 Name, physicochemical properties, description, strength, composition, usage, packaging and storage of study drug

Drug name

Common name: Icotinib Hydrochloride

Chemical name:

4-[(3-ethynylphenyl) amino]-6,7-benzo-12-crown-4-quinazoline hydrochloride

Structural formula:

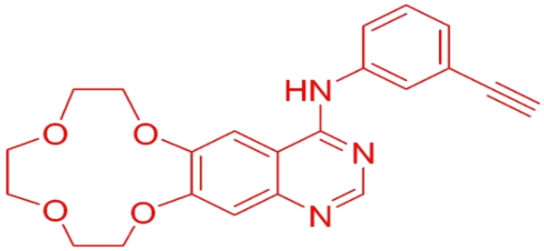

Molecular formula: $C_{22}H_{21}N_3O_4 \cdot HCl$

Molecular weight: 427.88

Physicochemical properties

Physicochemical properties: Icotinib hydrochloride is an odorless off-white to light

yellow cystalline powder without hygroscopicity. Soluable in dimethyl sulfoxide, it is dissolved slightly in acetonitrile: water (1:1), methanol or chloroform, extremely slightly in ethanol and virtually insoluable in water and acetonitrile. Melting point is 225-228℃. At a wavelength of 340nm, absorption coefficient ($E_{1cm}^{1\%}$) is 500-520.

Drug properties: Icotinib hydrochloride tablet is supplied as an oral formulation with a brown-red thin-layer coat. After decoating, it is of an off-white color.

Drug strength: For this trial, only the strength of 125 mg at a diameter of 11 mm is used.

Drug composition: Besides a major ingredient of icotinib hydrochloride, tablet core excipients include lactose, microcrystalline cellulose, croscarmellose sodium, polyvinylpyrrolidone K30 and magnesium stearate; excipients of coating agent include titanium dioxide, hydroxypropyl methyl cellulose and sedian.

Drug usage: It is indicated for treating advanced NSCLC.

Drug package: Inner package is made up of PVC press-through-package while external package is placed in a white high density polyethylene (HDPE) medicinal bottle with a white child-proof cap.

Drug storage: Stored under 25°C. Permitted temperature range is 15°-30°C. Shade from light and seal tightly.

9.2 Name, physicochemical properties, description,strength, composition, usage, packaging and storage of clinical control drug

Drug name

Common name: Gefitinib

Chemical name:

N-(3-chloro-4-fluorophenyl)-7-methoxy-6-(3-morpholinylpropoxy) quinazolin-4-amine

Structural formula:

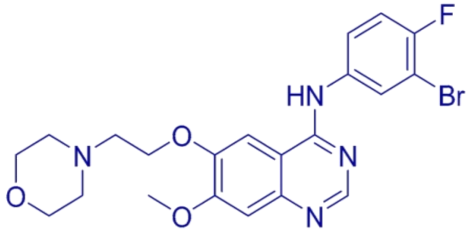

Molecular formula: $C_{22}H_{24}ClFN_4O_3$

Molecular weight: 446.90

Drug physicochemical properties

Physicochemical natures and characteristics: gefitinib is of while cystallaline powder. It is slightly soluable in aquous solution at pH 1 and virtually insoluable in aquous solution at pH 7. And at pH4-6, its soluability declines rapidly. It is dissolved easily in acetic acid and dimethyl sulfoxide. And it is soluable in pyridine, lightly in tetrahydrofuran and slightly in methanol, ethanol (99.5%) , ethyl acetate, isopropanol and acetonitrile. Melting point is 194-198 ℃.

Drug description: Tablets gefitinib is supplied as an oral formulation with a brown-red thin-layer coat. After decoating, it is of an off-white color.

Drug strength: Only one strength of 250 mg tablets with a diameter of 11.5 mm is used.

Drug composition: Besides a major ingredient of gefitinib, tablet core excipients include lactose monohydrate, microcrystalline cellulose, croscarmellose sodium, polyvinylpyrrolidone, sodium lauryl sulfate and magnesium stearate; excipients of coating agent include hydroxypropyl methyl cellulose, polyethylene glycol300, titanium dioxide, red ferric oxide and yellow ferric oxide.

Drug usage: Indicated for treating local advanced or metastatic NSCLC receiving previous chemotherapy or not suitable for chemotherapy.

Recommended dose of tablets gefitinib (250 mg) for adults: 250 mg, #1, po, qd, on empty stomach or taken with food.

In an event of dysphagia, tablets may be dispersed into half a cup of drinking water (non-carbonated drink). No other liquid should be used. Throw tablets into water. No crushing is needed. It is agiated until complete dispension (around 10 min). Wash down instantly the drug solution. Half a cup of water is used for rinsing. Drink the rinsing water. Also it is possible to feed through a nasogastric tube.

Dose adjustment is unnessary based upon the following situations: age, BW, sex, ethnicity, renal function, moderate-severe hepatic functional impairments caused by

hepatic metastasis.

Dose adjusting: In an event of untolerable diarrhea or skin AE, it is possible to resolve through a short period of therapy pausing for a maximal number of 14 days. Then a daily dose of 250 mg is resumed.

Drug package: Inner package is made up of PVC/aluminum foil blister.

Drug storage: Stored under 30°C.

9.3 Drug package and labeling

Icotinib hydrochloride tablets are packaged in a white high density polyethylene (HDPE) bottle with a white child-proof cap.

Study drug is packaged in a pre-labeled bottle. Each bottle label has the following items: protocol number, randomization number, batch number, dosing instructions and sponsor’s address.

To ensure the stability of icotinib hydrochloride tablets, drugs in original HDPE bottles should never be re-dispensed for useage.

9.4 Drug dispensing and inventory

In accordance with the standard operating procedures of Zhejiang BetaPharma Co., Ltd, the study drugs are delivered to each trial site. The study drugs are stored according to the protocol instructions.

Study drug should be dispensed by trial staffs.

After a proper interval or at the end of trial, all unused drugs and empty containers should be returned to Zhejiang BetaPharma Co., Ltd. And trial monitor should verify all unused supplies.

An inventory checklist must be provided and examined by a trial monitor. The supplied study drugs, including used or empty bottles and daily dispensing log, should be verified by a trial monitor.

10 Subject therapy

10.1 Study drug

Experimental group:

Icotinib 125 mg tablets, po 1# tid; gefitinib 250 mg placebo, po 1# qd until PD or intolerable toxicity.

Control group:

Icotinib 125 mg placebo, po 1# tid; gefitinib 250 mg tablets, po 1# qd until PD or intolerable toxicity.

Method of administration:

Both gefitinib and dummy tablets should be taken on an empty stomach at 1h pre-breakfast once daily. And tablets icotinib and dummy are consumed at #1 every 8h.

10.2 Concomitant medication

10.2.1 Prohibited medication prior to trial

- Use of Iressa, Tarceva, Erbitux and Herceptin as anti-neoplastic therapy prior to trial enrollment.
- Use of unapproved drugs or other trial agents during 4 weeks prior to the first day of study drug dosing.

10.2.2 Permitted and prohibited concomitant medications during the study

10.2.2.1 Concomitant therapies and smoking

All concomitant medications and therapies (including start/end dates & indications) should be recorded into the corresponding parts of source documents and CRF.

Gefitinib is greatly influenced by drugs metabolizing by hepatic enzymes. The effects of concurrent intakes of drugs inhibiting or inducing these metabolizing enzymes upon the in vivo metabolism of gefitinib have remained unknown. Thus the occurrences of AEs should be closely monitored when the above enzyme-modifying concomitant medications are taken.

Smoking affects the pharmacokinetics of gefitinib. During the course of therapy, patient smoking status should be recorded, including daily number of cigarettes and smoking duration. All patients on therapy are recommended to quit smoking.

10.2.2.2 Prohibited medications

- Prior to enrollment into the present trial, previous use of Iressa, Tarceva, Erbitux and Herceptin for anti-neoplastic therapy;

- Except for study drug, no other antitumor drugs, including experimental drugs of antibiotics and antiemetics, are allowed;
- Radiotherapy (except for palliative radiotherapy for existing bone metastatic lesions);
- During the course of therapy, any herbal antitumor therapy.

10.2.2.3 Permitted medications

- Non-conventional therapy, such as non-antitumor herbal therapy or acupuncture, use of vitamins/trace elements are at the discretion of the investigator as long as the observations of trial endpoint are not interfered;
- A patient may receive palliative and supporting therapies for existing disease, such as bisphosphonate and pain-killer;
- For symptomatic relief, radiotherapy for existing bone lesions (existing bone metastasis during baseline period);
- For those patients with existing bone metastatic lesions during baseline period in need of radiotherapy, it is best to complete radiotherapy prior to the first dose of study drug;
- For those patients with existing bone metastatic lesions during baseline period in no need of radiotherapy prior to the first dose of study drug therapy, radiotherapy is permitted if needed during the course of trial;
- For those patients with existing bone metastatic lesions undergoing radiotherapy during baseline period, radiotherapy is permitted if needed during the course of trial.

10.3 Subject compliance

For each subject, the dose of icotinib/gefitinib and medication time should be recorded on the CRF. Also the cause of delayed dosing, dose reduction or missed dose will be included.

Subject compliance to treatment and protocol include voluntary adherence to all aspects of protocol and collection of all blood samples for safety evaluations. When a subject fails to follow the visit or medication schedule, he/she may be removed from the trial at the discretion of the PI or sponsor.

Compliance to medication schedule: based upon the quality of drug dispensing and collection to calculate dosing status and considering the daily log card:

$$\text{Compliance} = \frac{\text{Actual dose taken}}{\text{Dose that should have been taken}}$$

If subject compliance is < 80％ or > 120％, the compliance is poor; if compliance < 70％, then the subject will be excluded from PP analysis. However such a case should be included in ITT analysis.

11 Efficacy evaluation

The objective is to compare the safety and efficacy of icotinib with gefitinib.

Primary efficacy variable is to compare the PFS between two groups.

Secondary efficacy variables include OS, ORR, DCR, TTP and lung cancer symptoms and HRQoL.

11.1 Primary efficacy variable

Progression-free survival (PFS): PFS is defined as duration from the date of randomization to the initial documentation of PD (as judged by imaging studies). For the patients who die from other causes prior to PD, PFS will be calculated as the days starting from the date of randomization to death. Tumor progression is defined as an increase of LD sum of measured lesions by at least 20% from the initiation of therapy as referenced by the minimal LD, or the occurrence of one or more new lesions. Occurrence of any new lesions indicates PD. Under special circumstances, a definite progression of non-measurable lesions may also be acceptable as the evidence of PD. An independent data monitoring committee will take charge for determining whether or not it is an imaging progression. For the progression-free patients survived up to the analysis date, the cutoff time is the date of their last imaging assessment.

11.2 Secondary efficacy variables

Overall survival (OS) is defined as the duration starting from the date of randomization to death from any cause. For the patients survived up to the analysis date, the cutoff time is the date of last contact.

Time to progression (TTP): It refers to the time starting from randomization to tumor progression. Tumor progression is defined as an increase of LD sum of measured lesions by at least 20% from the initiation of therapy as referenced by the minimal LD, or the occurrence of one or more new lesions. Occurrence of any new lesions indicates PD. Under special circumstances, the definite progression of non-measurable lesions may also be acceptable as the evidence of PD.

Tumor response and PD are evaluated by the investigator in accordance with the RECIST criteria.

HRQoL and lung cancer symptoms will be evaluated by the patient responses to the questions of FACT-L version IV and self-rated lung cancer symptom scale (LCS).

12 Safety assesement

The applicable population of safety analysis is made up of the patients who have received at least one dose of study drug. The results of physical examinations, vital signs, AEs and laboratory abnormalities will be summarized. All AEs should be reported and graded according to the NCI Common Terminology Criteria for Adverse Events (CTCAE) version 3.0 (NCI-CTCAE 3.0). However the grading of skin rashes associated with icotinib and gefitinib follows the grading criteria of EGFR-TKI-related skin adverse events.

12.1 Safety variables

12.1.1 Vital signs and physical examination

The patient will receive comprehensive physical examinations, including ECOG score, neurological examinations, height, BW, HR, BP, temperature and RR, etc.

12.1.2 Laboratory examinations

Routine blood biochemistry, urinalysis and pregnancy test will be conducted during screening period at the clinical laboratories of all study sites.

For the present trial, the following laboratory parameters will be evaluated.

Biochemical parameters (serum gel tube)

Alanine transaminase (ALT)
Aspartate transaminase (AST)
Alkaline phosphatase (ALP)
Total bilirubin (TB)
Total protein (TP)
Albumin (ALB)
Blood urea nitrogen (BUN)
Creatinine (Cr)
Potassium ion (K)
Sodium ion (Na)
Calcium ion (Ca)

Blood cell count (ethylenediaminetetraacetic acid [EDTA] tube)

Absolute neutrophil count (ANC)
Hemoglobin (Hb)
White blood cell (WBC)
Platelet count (PLT)

12.2 Adverse events

The occurrences of AEs must be closely monitored, including clinical laboratory examinations. The evaluation will be based on the grade, severity of the AE and its relationship with the study drug.

The investigator is in charge of evaluating the relationship between AEs and study drug. However the investigator may entrust other qualified clinicians participating in the present trial to make judgments, provided that the investigator remains accountable. A list of entrusted qualified clinicians must be provided by the investigator.

12.2.1 Definition of AE

AE refers to the occurrence of unexpected medical condition or a worsening of existing medical condition during the course of drug dosing, whether or not it is related with the study drug. Unexpected medical conditions may be symptoms (e.g. nausea & chest pain), signs (e.g. tachycardia & hepatomegaly), or abnormal test results (e.g. laboratory examinations & ECG). During the course of a clinical trial, after the signing of an informed consent form, AE may arise at any time as an unexpected adverse medical condition, even during the screening or washout period when the study treatment is not administered.

AEs occurring in humans (whether drug-related or not) include the following aspects:

- AE occurring during the administration of a drug by a professional;
- AE caused by drug overdose (whether intentional or unintentional);
- AE caused by drug abuse;

- AE caused by drug withdrawal;
- AE caused probably by sheer patient participation (e.g. AE or SAE caused by withdrawal of anti-hypertensive agent during washout period), even if unrelated with study drug.

If already documented on the appropriate part of CRF, the absence of or less than expected pharmacological effects should not be treated as AEs. If fulfilling the criteria of SAEs, they should be recorded and reported as SAEs. For the present trial, **any event caused specifically by PD should not be reported as AE.**

12.2.3 Unexpected AEs

Unexpected AEs are defined as any characteristic or severity which is inconsistent with the description of Investigator’s Brochure (or package insert of a marketed product). Supplementing the important information on the characteristics or severity of known and recorded AEs is also a component of unexpected AE. For example, events that are more special or more severe as compared with the description in Investigator’s Brochure should be deemed as “unexpected”. Specific examples include (a) acute renal failure indicated as AE and later developed into interstitial rhinitis; and (b) hepatitis initially reported as acute necrosis.

12.2.4 Observation, recording and reporting of AEs

All AEs occurring after signing informed consent form should be recorded completely on the CRF.

The entries must be supported by source documents. Each event should be described in details, including start/end date, severity, correlations with study drugs, actions taken and outcome.

12.2.4.1 Methods of identifying AEs

At each visit, the following methods may be employed to identify AEs:

- Information provided by a patient or guardian without any prompt;
- At each visit, asking open-ended and non-suggestive questions such as “How do you feel?” “Since the last visit, have you had any medical or other issue?”

- Abnormalities observed by the investigator, other medical professionals and relatives.

12.2.4.2 The period for Collecting AEs

For the present study, non-severe AEs will be collected from the date that patient signing informed consent up to the 30-day follow-up period following discontinuation of treatment.

12.2.4.3 Collection of AE data

All AEs should be recorded on the CRF. The description of AEs will include start/end time, whether it is serious or not, actions taken (e.g. study treatment modifications, other therapies and subsequent examinations) and outcome, and the investigator will be requested to evaluate the causality (correlation with study treatment). AEs should be graded according to the criteria of NCI-CTCAE 3.0 and their changes will be recorded on the CRF.

12.2.5 Evaluation for AE severity

The following grading criteria will be adopted:

The severity of AEs should be recorded in accordance with the Common Terminology Criteria for Adverse Events (CTCAE) version 3.0 (NCI-CTCAE 3.0).

12.2.6 Relationship between AEs and study drug

In accordance with the national *Measures for the Reporting and Monitoring of Adverse Drug Reactions*, the correlation between the adverse reaction/event and the study treatment may be graded as definitely related, probably related, possibly related, unlikely related, uncertain or unknown based on five criteria.

	**1**	**2**	**3**	**4**	**5**
Definitely	+	+	+	+	—
Probably	+	+	+	?	—
Possibly	+	±	±?	?	±?
Unlikely related	—	—	±?	?	±?
Uncertain	Supplemental materials required for evaluations				
I Unknown	Required evaluation materials are not available.				

Note: +: affirmative; －: negative; ±: affirmative or negative undetermined; ?: unknown

Five criteria for adverse reaction/event analysis:

1. Is there a reasonable temporal relationship between medication and the occurence of adverse reaction/event?
2. Is the reaction consistent with the known adverse reactions for this drug?
3. Does the reaction resolve or relief after treatment interruption or dose reduction?
4. Does the same reaction/event recur when the suspicious drug is resumed?
5. Can the reaction/event be explained by the effects of concomitant medication, PD or other therapies?

If considered as definitely, probably or possibly related, the event should be treated as a drug-induced adverse reaction, and may be considered as an SAE depending on its severity.

For the present trial, it is at the discretion of the investigator to determine whether a simple laboratory abnormality is clinically significant, and the abnormality may not be reported as an AE if it is not clinically significant. In case on an unexplainable laboratory abnormality, the test should be immediately repeated and followed up until it returns to normal range and/or is sufficiently explained. A definite explanation will be included in the CRF.

12.2.7 Treatment, follow-up and duration of AEs

After an initial report for AE, the investigator should conduct close follow-ups on the patient so as to provide further information to Tigermed. During the course of the trial (even if trial/therapy has completed), all AE should be followed up until resolution or stable, unless the AE is not possible to resolve due to existing condition based upon the investigator’s judgment or patient is lost to follow-up.

12.2.8 Common AEs of icotinib and gefitinib

Common AEs with icotinib include (incidence >10%) in early studies: skin rash, diarrhea; rare AEs: transient abdominal pain, nausea and abnormal transaminases.

An investigational drug though, gefitinib has been marked both home and abroad. The safety data from a large sample size are available.

Common AEs with gefitinib (incidence >10%): skin rash, diarrhea, fatigue and tachypnea, etc. And rare AEs include paronychia, hair & eyelash abnormalities and blood biochemical abnormalities, etc.

12.2.9 Determination and handling of laboratory abnormalities

Any laboratory abnormality should first be compared by the investigator with baseline value for assessment of its clinical significance, and only those AEs assessed as clinically significant with changes from baseline should be reported as AEs, and will be followed up until normalization or return to baseline value. All the abnormal laboratory test values/vital signs should be accurately recorded on appropriate CRF page.

12.3 SAEs

12.3.1 Defintion of SAE

An SAE is any AE that occurs at any study phase (i.e. screening, treatment or follow-up) during administration of the study drug, comparator drug or placebo at any dose and meeting one or more of the following:

- Fatal;
- Immediate life-threatening
- Require hospitalization or prolongation of existing hospitalization;
- Results in persistent or significant disability/incapacity or;
- A congenital anomaly/birth defect;
- Any important medical event that jeopardize the patient or may require medical intervention to prevent one of the other outcomes listed in the definition above.

Any event or hospitalization caused by the progression of a specific disease should not be reported as SAE. Any death caused definitely by PD must be recorded on the CRF. During the next visit, it will be reported to the monitor but **may not necessarily** be reported as SAE.

Life-threatening: The term "life-threatening" in the definition of "SAE" refers to an event in which the patient was at risk of death at the time of the event; it does not refer to an event which hypothetically might have caused death if it were more severe.

Hospitalization: Any AE leading to hospitalization or prolongation of existing hospitalization will be considered serious **unless** conforming to one of the following:

- Hospital observation is less than 12h;
- Hospitalization is previously planned (i.e. elective operation scheduled prior to the start of trial);

- Hospitalization is unrelated with AE (e.g. for health maintenance).

Disability indicates severe impairment of the ability of doing daily living activities

12.3.2 Important AE

A medical events that may not be considered serious but is considered by the investigator as unexpected and may be associated with potential risks that require medical intervention to prevent one of the other outcomes listed in the definition above.

12.3.3 Recording and reporting procedures of SAE / pregnancy

Reporting procedures of SAEs are as follows:

- Within 24h，the investigator reports SAE to the site ethics committee and fax it to Tigermed monitor ;
- Upon receiving an SAE report, Tigermed monitor faxs it to sponsor and other co-investigators within 24h;
- Upon receiving an SAE report, PI reports it to Central EC within 24h;
- Upon receiving an SAE report, PI and sponsor report it within 24h;
- Upon receiving an SAE report, sponsor reports it to Zhejiang Provincial FDA within 72h;
- Upon receiving an SAE report, each co-investigator reports it to his/her hospital EC as soon as possible;

For fatal or life-threatening events, if required and feasible, a copy of hospital records，such as any relevant diagnostic result, histopathological report, discharge summary, autopsy form, postmortem findings and other documents, should be faxed.

Beta Pharma may request the trial staff to provide other information to ensure the timely and accurate completion of a safety report.

The trial staff must take all necessary treatments to resolve the SAE. Any medication required for SAE treatment must be recorded in concomitant medication section of the patient’s CRF.

The follow-up of AE should be continued until the last day of the study (including follow-up and drug withdrawal periods), until the investigator considers the patient’s condition as stable, or until 30 days after last dose of study drug, whichever is longer.

12.3.4 Special treatments for SAEs

If interstitial lung disease is suspected upon clinical and auxiliary examinations, the

patient should receive lung biopsy to confirm the diagnosis (unless the patient declines).

If death occurs which is not due to a definite PD (except for accidental death), the investigator should contact the subject’s relatives. Autopsy is recommended to confirm the cause of death. If such a request is turned down by the subject’s relatives, the investigator should record it.

13 Data management

13.1 Data traceability, and CRF completion and transfer

As the original records, the study medical records should be properly stored. CRFs, sourced form of the study medical records, will be completed by the investigator. Every enrolled subject must complete a CRF. Upon review by the clinical monitor, a copy of the completed CRF will be submitted to the statistics unit for data entry and management. Upon the submission of the copy, CRF will no longer be revised.

13.2 Design and establishment of database

The trial database should be designed according to the data management plan, allowing the functions of double entry and verification, data trace ability and database lock. Prior to formal data entry, pre-entry will be performed and data safety and confidentiality will be tested.

13.3 Data coding

AEs and medical history are coded by Med DRA.

13.4 Data entry and revision

The data entry and management are carried out by the Statistics Department of Tigermed, which will assign special persons in charge of data management and planning. To ensure the accuracy of numeric type data, double entry and proof-reading will be adopted.

For any queries in the CRF, the data manager will complete the DRQ and direct the inquiries through a clinical monitor to the investigator. Queries should be answered and returned as soon as possible. Based upon the investigators’ feedbacks, the data manager revises, confirms and enters the data. If necessary, DRQ may be re-sent.

13.5 Data verification

The data manager will conduct blind data verification and issue a blind verification. PI,

sponsor, statisticians and data manager will jointly audit this report and finalize the statistical analysis sets before data locking.

13.6 Database lock and unblinding

After blind verification and establishment of a correct database, database lock will be performed jointly by PI, sponsor and statisticians. In principle, the locked database should no longer be modified. Any problem identified after data locking, once confirmed, will be revised within a statistical analysis program, and a written record will be maintained.

After database lock, unblinding will be performed by the staffs of the leading site which is responsible for maintaining the blind code, sponsor and statisticians to assure that each subject’s drug code corresponds to either experimental group or control group. And the database will be submitted to the statistical analysis staff for the pre-planned analyses. A Statistical Analysis Report will be drafted the by the statistical unit and submitted to the PI for the Clinical Study Report.

14 Statistics

14.1 Estimation of sample size

The primary efficacy variable of this trial is to compare the PFS of icotinib with gefitinib between the two groups. PFS is defined as the duration starting from the date of randomization until initial imaging evidence of PD. If death occurs prior to PD due to other causes, then the number of days is counted from the date of randomization until death.

The non-inferiority trial design is adopted for the present trial. An inter-group comparison of median PFS is used to complete the non-inferiority test. Since PFS has a non-normal distribution, log values are taken for analysis. According to an Asian meta-analysis, the median PFS of gefitinib was around 4.2 months. When compared with gefitinib, the expected median PFS is no less than 87.5% of that of gefitinib, i.e. 3.675 months. Assuming α＝0.05 (one-sided), β＝0.2 (power of test＝80%), non-inferiority threshold value＝87.5%, experimental group: control group＝1:1，coefficient of variability (CV) (S/X)＝0.5, sample size are at least 156 and 156 cases for either group. Considering a

dropout rate of 10%-15%, the present trial is projected to recruit 400 cases (200 cases for either group).

Since the sample size of test group falls short of the minimal sample size of 300 cases for a phase III clinical trial according to the SFDA guideline. After completing the pre-planned sample size of 200 pairs, we may perform an additional single group trial of at least 100 cases to fulfill the requirements of SFDA.

14.2 Definition and selection of analysis sets

For the present clinical trial, 3 statistical datasets are adopted:

Full-analysis set (FAS): All randomized subjects who have used at least one dose of the study drug and did not seriously violate to the protocol in anyway.

Per-protocol set (PPS): Subjects in the FAS who have used the study drug per protocol, not used prohibited medications, have no major protocol violations, and have discontinued the trial due to endpoint events (PD or death).

For efficacy analysis, both FAS and PPS are used. However the former is primary.

Safety set (SS): All randomized subjects who have used at least one dose of the study drug.

14.3 Hypothesis test

The primary efficacy variable is PFS. For an inter-group comparison, the COX proportional risk model will be employed. And the center effect is considered. The non-inferiority test of median PFS will be performed to demonstrate that the median PFS of experomental group is non-inferior to that of control group:

$$H_0 : \frac{\mu_T}{\mu_C} = \phi \le \delta \qquad H_1 : \frac{\mu_T}{\mu_C} = \phi > \delta$$

At a level of $\alpha = 0.05$ (one-sided), H_0 is refuted. Thus it is considered that test group is non-inferior to control group.

For other parameters, differential test is adopted for inter-group comparison ($\alpha = 0.05$, two-sided).

14.4 Statistical methods

14.4.1 Analytic methods

14.4.1.1 Baseline and demographic characteristics

Baseline data include demographics, baseline tumor characteristics, medical history, previous antitumor therapies, concomitant medications and vital signs, etc. Mean, standard deviation, median value, minimum and maximum are used for the description of continuous variables; frequency and percentage used for the description of categorical variables. The inter-group comparisons of baseline parameters will be made by group t test or Wilcoxon rank-sum test and Fisher's exact probability method.

14.4.1.2 Primary efficacy analysis

The primary efficacy variable is PFS.

PFS is defined as the number of days starting from the date of randomization until initial imaging evidence of PD. If death occurs prior to PD due to other causes, then the number of days is counted from the date of randomization until death. During calculations, actual time is used for tumor assessment. For those patients whose PD or death is absent during analysis, the cutoff date of PFS is the time of final tumor assessment. If no PSF is evaluated since the baseline period, the cutoff date of 1 day is used. The investigator's judgment is used to determine the progression time. The lost or invalid tumor assessments are used to the calculations of PFS.

For PFS, the Kaplan-Meier method will be employed to estimate the survival curves of two groups. And the COX proportional risk model is used for an inter-group comparison. Also center effect and important co-variates are considered.

Secondary efficacy analysis

Overall survival (OS): It is defined as the duration starting from the date of randomization until death from any cause. During the time of analysis, the cutoff date is the day of last contact. The statistical method is the same as that of PFS.

Overall response rate: All patients are assessed according to the RECIST criteria. Incomplete or partial tumor assessment are not used to calculate the response rate unless PD is present in lesions on the above examinations. Those receiving no evaluations of lesions and tumor are not calculated. The Fisher's exact probability method will be

employed for an inter-group comparison. The logistic regression model is used for multi-factorial analysis.

Patient self-rated measurements

Lung cancer symptoms are evaluated on the basis of patient responses to the LCS scale. LCS scale is validated to evaluate the effects of therapy upon lung cancer symptoms. This scale is composed of 7 questions. And the answer to each question is divided into 5 successive classes of “Never” to “Quite”. The total score of LCS scale is 0-28 points. A higher score indicates more marked lung cancer symptoms.

HRQoL is evaluated on the basis of patient responses to the FACT-L questionnaire. FACT-L questionnaire is validated to evaluate the QOL of lung cancer patients. This 36-item questionnaire includes the following 4 aspects: performance status, functional wellbeing, emotional wellbeing and social/family wellbeing. The answer options have varying degrees of from “Never” to “Quite”. The total score of FACT-L questionnaire is 0-136 points. A higher score indicates a worse QOL.

The total scores of LCS scale and FACT-L questionnaire are analyzed by the method of ANOVA. Within the model, baseline total score and stratification factors during the course of randomization are considered.

14.4.1.3 Safety analysis

In accordance with the Common Terminology Criteria for Adverse Events (CTCAE) version 3.0, the AEs and the severities will be summarized, and AEs will also be summarized based upon event severity and its relationship with study drug. The descriptive summaries of laboratory tests will be for the abnormal values. The laboratory abnormalities will also be summarized in accordance with the most severe grade in the NCI-CTCAE 3.0. For skin toxicities, refer to the consensus of skin rash in appendix.

14.4.2 Interim analysis

No interim analysis will be conducted for the present trial. However an Independent Data Monitoring Committee (IDMC) will be established. The members of IDMC include oncologists, imaging experts and one senior statistician. Internal meetings of IDMC will be held for the members to clarify their functions and duties, and to formulate the independent third-party adjudication criteria for the efficacy results judged by the

investigator. Upon the conclusion of the trial, the efficacy of all subjects will be independently judged by a third party.

15 Trial management

15.1 Statement

The present clinical trial will be conducted strictly in accordance with the specifications of the protocol, the guidelines for Harmonization/Good Clinical Practice (GCP), the Declaration of Helsinki, regulations and SOP requirements.

15.2 Ethnics considerations

The investigator is obliged to provide EC with clinical study protocol, informed consent form and subject's information sheets to obtain an independent approval document for the implementation of this clinical trial.

Prior to the start of this trial, the approval document of EC must be obtained and submitted in a written form to the investigator. Then a copy of approval document is provided to the sponsor. And the EC approval document should be attached with a list of all committee members participating in the discussions of approval document and their respective duties.

During the course of the trial, any question related with safety, such as modifications of clinical trial protocol or informed consent form and occurrences of SAE, should be timely reported to EC. The conclusion or premature termination of clinical trial should also be reported to EC.

15.3 Source data verification

For direct entries onto the CRF (i.e. no previous written or electronic copy) and for identification of source data, it should be pre-specified within the monitoring plan in accordance with the protocol. Unless definitely specified, a lack of source data will be considered.

The investigator must properly process all data acquired throughout the trial to guarantee the rights and privacies of subjects. A monitor/auditor/inspector must obtain the permission of the investigator to review and examine the relevant data so as to verify the accuracy of source data and to acquire the progression of the trial. If source documents

can not be verified, the investigator should agree to assist a monitor/auditor/inspector in further confirmations of data quality.

15.4 Quality control and assurance

In the present clinical trial, quality control must be enforced for all drugs and materials. The sponsor-entrusted staff or relevant medical regulatory authorities have the right to audit the clinical trial. The purpose is to ensure the reliability of the data and the observance of protocol provisions. The subjects will be informed that the relevant personnel may perform audit during the course of trial, but their privacy and data will be strictly protected.

15.5 Informed consent form/data protection agreement

The investigator is obliged to explain the objectives, method, benefits and potential risks of this trial to each subject and obtain an informed consent form signed by the subject. Prior to the start of any trial-related procedure, a subject’s informed consent form must be obtained. If a subject fails to sign an informed consent due to any cause, their parent, legal guardian or protecting person must do so on his or her behalf. Through the signing of an informed consent form, a subject must also agree to allow the monitor/audit/health surveyor of this trial to verify the collected source data so as to ascertain the reliability of data.

The original copy of informed consent form signed and dated by participants must be properly stored by the investigator. The signing of an informed consent formed should also be recorded on the CRF and trial-related source documents.

15.6 Revision of clinical protocol

The revision of clinical protocol must be jointly made by the sponsor and investigators. The protocol revision should be submitted to EC for review and approval. After approval, the revised contents may be implemented.

15.7 CRF

The investigator should fill in CRF completely and accurately. Each CRF records the information of only one subject. All data or texts filled in incorrectly should never be altered. Instead they must be marked with a single line. Then the correct data or texts are displayed next to it. And the investigator signs his or her name and the correction date.

The original copy of CRF is maintained by the sponsor and photocopies are filed at each study site. For data entry, the original copy of CRF is required.

15.8 Trial monitor

A trial monitor is obliged to familiarize with PI, co-investigators (including dispensing study drug) and nurses participating in the trial. Monitors must regularly monitored the trial progression at each site: at the enrollment time of the first trial subject, during the course of trial and during different phases after the end of trial.

A monitor is obliged to cooperate with the investigator to verify whether or not the trial is implemented strictly in accordance with the clinical study protocol, and jointly resolved any issue occurring during the course of trial with the investigator. Also a monitor should drouble-check the records of clinical trial and source document to ensure the truthfulness of clinical files.

A monitor should follow standard operating procedures to offer a detailed account of source data verification. The informed consent forms of all enrolled trial subjects must be verified.

15.9 Subject privacy

The trial staff must safeguard the trial subject's privacies. In all documents and files submitted to sponsor, the trial subjects can only be identified by patient number and initials. Full name of a subject is never used. The investigator should properly maintain the name, address and corresponding enrollment form of a trial subject. The enrollment form is maintained by the investigator with strict confidentiality and may not be submitted to the sponsor.

15.10 Publications

Prior to the publication of the entire multi-center trial results, each center should not publish its results separately.

Prior to the publication or publicity of trial results, sponsor has the right to review the draft.

15.11 Filing

In accordance with the relevant regulations, the investigator should properly maintain the source documents of this clinical trial. All clinical trial files must be maintained for 15 years according to the requirements of ICH GCP.

16 References

1 Icotinib Investigator’s Manual.

2 Wei Z, Mengzhao W, Li Z, Longyun L, Evaluation of efficacy and safety of gefitinib as monotherapy in Chinese patients with advanced non-small cell lung cancer and very poor performance status. BMC Res Notes 2008 Oct 28; 1:102.

3 Bunn PA Jr, Thatcher N. Systemic treatment for advanced (stage IIIb/IV) non-small cell lung cancer: more treatment options; more things to consider. Conclusion. Oncologist. 2008; 13 Suppl 1:37-46.

4 Gridelli C, Bareschino MA, Schettino C, Rossi A, Maione P, Ciardiello F Erlotinib in non-small cell lung cancer treatment: current status and future development. Oncologist 2007 Jul;12(7):840-9.

5 Sequist LV. Second-generation epidermal growth factor receptor tyrosine kinase inhibitors in non-small cell lung cancer. Oncologist, 2007 Mar; 12(3).

17. Protocol signature page

Investigator's statement

I have read through the protocol. The study shall be conducted in accordance with the moral, ethnic and scientific guidelines of the Helsinki Declaration and Chinese GCP. I agree to conduct this clinical study according to the protocol designs and specifications.

I shall be responsible for making the medical decisions related with this clinical study and will ensure that the subjects are timely treated for any AE occurring during the study period. I know the proper procedures and requirements of reporting SAE and will record and report these events based on these requirements

I shall promise to enter the study data accurately, completely, timely and legally on the CRF. And I accept the inspections or audits of a monitor or auditor delegated by sponsor or drug regulatory departments to ensure the quality of this clinical study.

I agree to use the study results for drug registration.

Prior to the start of study, I shall submit a CV to the ethnics committee or probably the competent drug regulatory administration.

Investigator (signature): ______________________ Date: ______________
